# Loss of novel mechanosensitive ion channel TMEM63A impairs osteogenic differentiation and proliferation via disruption of cellular mechanotransduction

**DOI:** 10.1016/j.mbm.2026.100212

**Published:** 2026-09-09

**Authors:** Meghana Davuluri, Rekha Sathian, Anna R. Cho, Ramana V. Davuluri, Yi-Xian Qin

**Affiliations:** a Department of Biomedical Engineering, Stony Brook University, New York, United States; b Department of Biomedical Informatics, Stony Brook University, New York, United States

**Keywords:** TMEM63A, Mechanotransduction, LIPUS, Osteoblast differentiation, Wnt/β-catenin, Calcium signaling, Mechanosensitive ion channel, RNA sequencing, Bone cell mechanobiology, Osteoblastogenesis

## Abstract

Skeletal homeostasis depends critically on the local mechanobiological microenvironment. While well-known mechanosensitive ion channels like Piezo1 and Piezo2 translate physical forces into immediate membrane ion fluxes, the proximal sensors regulating medium- and long-term osteogenic pathways remain poorly understood. Low-intensity pulsed ultrasound (LIPUS) is widely used clinically to accelerate fresh fracture repair, yet its underlying upstream mechanotransduction machinery is not fully characterized. Here, we identify TMEM63A – a stretch-activated cation channel – as a crucial mechanosensor required for mechanical stimuli-driven osteogenic differentiation and anabolic signaling for sustained response to the force. Thus, the **objective of this study was to elucidate the function of TMEM63A in driving osteogenic differentiation and modulating ultrasound-driven anabolic signaling during mechanotransduction with both short- and long-term effects**. In MC3T3-E1 pre-osteoblasts, stable shRNA-mediated knockdown (KD) of TMEM63A (∼63% mRNA and ∼60% protein reduction) significantly impaired osteogenic capacity. KD cells exhibited reduced alkaline phosphatase activity at Days 7 and 14, suppressed proliferation, attenuated early marker expression (Runx2, Alp, and Col1a1), and a marked reduction of LIPUS-evoked Ca^2+^ oscillations and nuclear β-catenin translocation. Genome-wide RNA sequencing revealed coordinated downregulation of bone formation, mineralization, and ossification networks in KD cells across Days 7 and 14. Gene set enrichment analysis (GSEA) demonstrated negative enrichment for Hallmark Wnt/β-catenin and PI3K-AKT-mTOR signaling pathways at Day 14, paired with positive enrichment for matrix-degradation and remodeling programs, consistent with *Mmp13* upregulation. Crucially, LIPUS treatment failed to rescue or engage these osteogenic programs in TMEM63A-deficient cells. Under Day 14 LIPUS, knockdown cells maintained negative enrichment of Hallmark Wnt/β-catenin signaling and Reactome Axin degradation, a positive readout of canonical Wnt activation, indicating that the β-catenin destruction complex remains active and continues to target β-catenin for degradation. Taken together, these findings establish TMEM63A as an essential mechanosensor that couples acoustic force stimulation via LIPUS to a Ca^2+^ → AKT → GSK3 β → β-catenin signaling axis to drive osteogenic transcription and bone formation through cellular mechanotransduction.

## Graphical abstract

**Figure:**
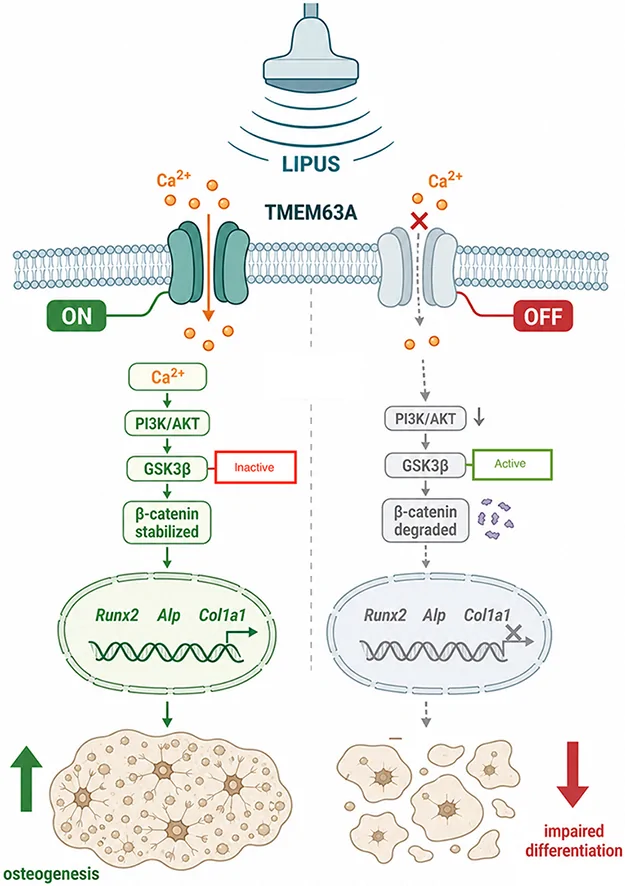

## 1 Introduction

The skeleton continuously senses and adapts to its mechanical environment. Physiological loading generates extracellular matrix strain and interstitial fluid flow, which osteolineage cells transduce into intracellular signals that control proliferation, differentiation, and remodeling. This conversion of physical cues into biochemical activity, termed mechanotransduction, stabilizes bone mass and architecture through coordinated actions of osteoblasts, osteocytes, and osteoclasts. A convergent set of signaling axes mediates these effects, prominently Wnt/β-catenin, YAP/TAZ, MAPK/ERK, and PI3K/AKT pathways, with pathway crosstalk and temporal coding that reflect stimulus magnitude and frequency.^1^ Recent studies on Piezo1 and Piezo2 reveal how changes in membrane ion channel activity can rapidly disrupt cellular signaling.^2^^,^^3^ However, the signaling pathways that regulate medium- and longer-term cellular activation remain poorly understood.

Unbiased transcriptomics has provided high-resolution insight into how these signaling pathways are temporally orchestrated during osteoblast maturation. Longitudinal bulk RNA-seq of MC3T3-E1 pre-osteoblasts across seven time points (Days 0, 1, 4, 7, 10, 14, and 21) identified three distinct and sequential transcriptional stages, proliferation, matrix maturation, and mineralization, and revealed a positive correlation between proliferation-associated and ossification-enriched gene programs during the mineralization stage, challenging a strictly sequential model of osteoblast development.^4^ Complementary microarray transcriptomic profiling of MC3T3-E1 cells across four maturation stages (Days 2, 5, 10, and 28) further demonstrated temporally coordinated activation of Wnt/β-catenin signaling during the mid-differentiation phase (Days 5-10), followed by PI3K/AKT and MAPK pathway enrichment during maturation (Days 10-28), with BMP and TGF-β signaling active throughout.^5^ These datasets collectively establish that Wnt/β-catenin and its converging kinase pathways are not merely permissive but required during specific differentiation windows, a framework that directly informs the interpretation of mechanically regulated gene programs.

**Fig. 1:**
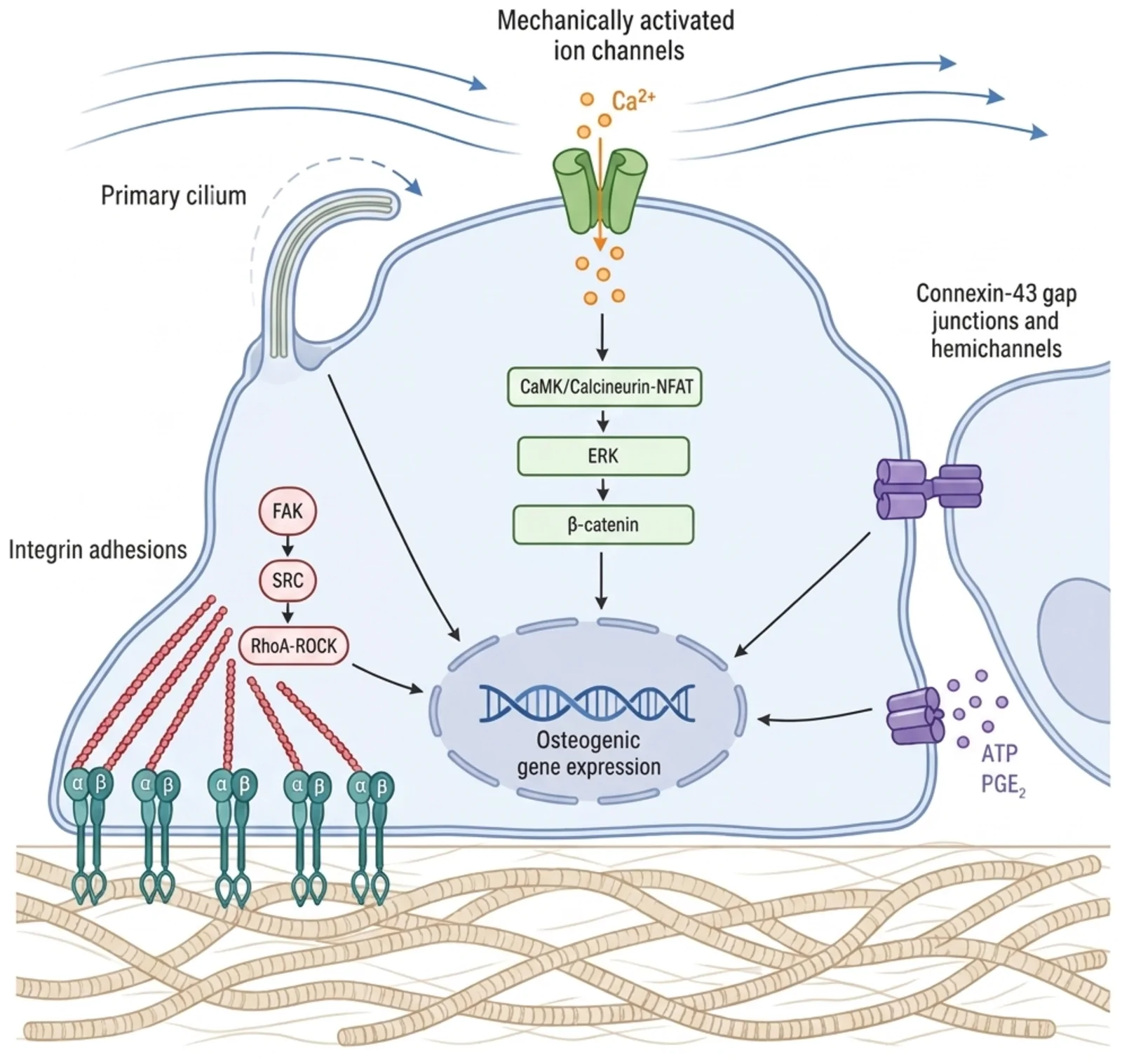
**Parallel mechanosensory modules in osteoblasts converge on osteogenic transcription.** Integrin-based adhesions couple the extracellular matrix to the actin cytoskeleton and activate FAK, SRC, and RhoA-ROCK signaling. Primary cilia deflect in response to interstitial fluid shear. Connexin-43 gap junctions coordinate multicellular responses to loading while hemichannels mediate paracrine ATP and PGE_2_ release. Mechanically activated cation channels open under membrane tension, producing Ca^2+^ influx that engages CaMK/Calcineurin-NFAT, ERK, and β-catenin signaling. These modules act in parallel and converge on shared signaling nodes to specify osteogenic gene expression. Created using BioRender (https://BioRender.com).

Mechanosensation in bone is distributed across several structures that operate in parallel (Fig. 1) and feed common signaling nodes. (i) Integrin-based adhesions couple the extracellular matrix to the actin cytoskeleton; force loading activates focal adhesion kinase (FAK) and SRC, reinforces stress fibers via RhoA-ROCK, and supports load-induced anabolic responses in vivo.^6^, ^7^, ^8^ (ii) Primary cilia, solitary microtubule-based organelles, bend under fluid shear and contribute to developmental and adult mechanotransduction programs in osteolineage cells.^9^^,^^10^ (iii) Connexin-based gap junctions and hemichannels, particularly connexin-43 (Cx43), coordinate multicellular responses to loading/unloading and mediate paracrine ATP and PGE_2_ release.^11^^,^^12^ (iv) Mechanically activated ion channels open with membrane tension and shear, producing rapid Ca^2+^ influx that triggers CaMK/Calcineurin-NFAT, ERK, and β-catenin signaling.^2^ These modules integrate cytoskeletal tension, nuclear mechanics, and second-messenger dynamics to specify osteogenic gene expression.

Among mechanically activated ion channels, PIEZO1 is a well-validated bone mechanotransducer.^13^ PIEZO1 and PIEZO2 were identified by Coste and colleagues in 2010 as essential components of distinct mechanically activated cation channels.^14^ Cryo-electron microscopy structures revealed that PIEZO channels form trimers with a propeller-like shape that bends the local lipid bilayer, resulting in an area of inherent curvature that may be controlled by membrane tension.^15^^,^^16^ Osteoblasts, which are responsible for bone matrix formation, are derived from mesenchymal stem cells (MSCs) in the bone marrow stroma. Their differentiation follows a clear hierarchy: MSC to osteoprogenitor to pre-osteoblast to mature osteoblast, with distinct transcription factor expression indicating each stage. Runt-related transcription factor 2 (Runx2) is the earliest osteogenic commitment marker and is necessary for osteoblast lineage commitment; Runx2-deficient mice do not generate bone.^17^ Osterix (Osx, also known as Sp7) functions downstream of Runx2 and is essential for the transition from preosteoblasts to mature osteoblast.^18^ Genetic activation of Piezo1 in osteoblast-lineage cells increases bone formation, whereas Piezo1 deletion reduces bone mass and blunts osteoblast-osteoclast crosstalk, establishing that force-gated channels can function upstream of canonical osteogenic pathways.^19^, ^20^, ^21^ Notably, ∼43% of mechanically activated current persists in Piezo1-knockout osteoblasts, implying that additional, unidentified mechanosensitive channels contribute to osteoblast Ca^2+^ influx.^19^ Piezo1/2 double knockouts produce more severe bone phenotypes than either single knockout, further demonstrating functional redundancy and the existence of parallel force-sensing mechanisms.^22^ Resolving the identity and downstream signaling contributions of these residual mechanosensors represents a key open question in skeletal mechanobiology.

Low-intensity pulsed ultrasound (LIPUS) provides a noninvasive, precise mechanical stimulus to cells and tissues. LIPUS is FDA-cleared for fresh fracture healing and nonunions, and delivers a non-thermal acoustic radiation force stimulus to osteoblasts and osteocytes.^23^ Acoustic radiation force and acoustic microstreaming generate nanoscale membrane displacements and localized shear that can activate integrins and mechanosensitive channels, engaging ERK/MAPK and PI3K/AKT signaling.^3^ Bulk RNA-sequencing of LIPUS-treated rat bone marrow mesenchymal stem cells (BMSCs) has revealed that LIPUS-upregulated genes are significantly enriched for the Gene Ontology biological process term “osteoblast differentiation,” with random forest analysis identifying ITGA11 (Integrin α11) as the dominant differentially expressed gene driving osteogenic outcomes. Pathway validation confirmed that LIPUS activates a FAK → PI3K → AKT → GSK3β → β-catenin signaling cascade downstream of ITGA11 upregulation, directly implicating integrin engagement in Wnt pathway activation.^24^ In vitro and preclinical studies in osteoblasts and mesenchymal progenitors report increased proliferation, migration, alkaline phosphatase (ALP) activity, and osteogenic gene expression after LIPUS exposure, frequently through ERK-linked mechanisms.^25^, ^26^, ^27^ RNA-seq of LIPUS-treated osteocytes further identified immediate-early transcription factor responses, including FoxQ1 (Wnt/TGF-β-linked cell proliferation regulator) and Helz2 (PPARγ coregulator coupling bone formation to resorption), within the first minutes to hours after stimulation,^28^ suggesting that LIPUS drives a rapid transcriptional cascade upstream of the differentiation-stage-specific programs documented by longitudinal transcriptomics. However, clinical efficacy data are mixed; a large, randomized trial in operatively managed tibial fractures found no improvement in radiographic healing or function with postoperative LIPUS,^29^ and a systematic review found that only trials at high risk of bias reported benefit.^30^ These inconsistencies underscore the need to define the cellular mechanosensors and parameter dependencies of ultrasound mechanotransduction, particularly the upstream channels that link acoustic stimulation to intracellular signaling.

TMEM63A belongs to the conserved OSCA/TMEM63 family of stretch-activated cation channels. Biophysical and structural studies indicate that TMEM63 proteins form membrane-embedded pores gated by bilayer tension, functioning as monomeric, high-threshold, slowly inactivating channels with non-selective cation permeability and robust activation by mechanical stimuli.^31^^,^^32^ Channel inactivation kinetics refers to the time at which a channel closes in response to a continuous stimulus. Mechanosensitivity of TMEM63A specifically is assessed by membrane stretch rather than by indentation, since TMEM63A produces no measurable currents in response to whole-cell poking and is activated only by negative pressure applied to a membrane patch.^32^ Its activation threshold is therefore expressed as the pressure required for half-maximal activation, P_50_. The minimal pressure required for half-maximal activation (P_50_) of mouse TMEM63A was found to be ∼ −60 mmHg, while the P_50_ value for mouse PIEZO1 was ∼ −24 mmHg.^32^ TMEM63A also activates approximately fourteen-fold more slowly, with time constants of ∼126 ms and ∼9 ms, respectively, and inactivation tau constant is ∼323 ms and ∼28 ms respectively. Inactivation, meaning channel closure during a continued stimulus, is likewise at least an order of magnitude slower than PIEZO1, and several studies report that TMEM63A currents do not inactivate appreciably at all.^31^, ^32^, ^33^ TMEM63A is also gated as a monomer, whereas PIEZO1 assembles as a trimer.^31^ TMEM63A remains largely closed under the small deformations sufficient to gate PIEZO1. Unlike PIEZO1 or the polymodal TRPV4, this combination of a high activation threshold, slow activation, and slow or absent inactivation positions TMEM63A to conduct throughout a sustained or repetitive mechanical stimulus rather than reporting only its onset, a property relevant to the pulsed acoustic radiation force delivered by LIPUS. We consider the implications of these kinetics for signaling across a single exposure, and their relationship to differentiation measured over days, in the Discussion. Despite these biophysical properties, TMEM63A's role in the osteoblast lineage has not been defined. Proteomic analysis of human bone marrow stromal cells (BMSCs) identified TMEM63A among the ten most downregulated proteins under simulated microgravity, with the authors proposing that reduced TMEM63A expression decreases Ca^2+^ influx and contributes to impaired osteogenic differentiation.^34^ Whether TMEM63A contributes to mechanosensory Ca^2+^ entry in osteoblasts, and how it shapes downstream osteogenic signaling, remain open questions.

Given the established mechanosensory roles of integrins, Cx43, primary cilia, and PIEZO1, and the ∼43% of mechanically activated current unaccounted for in Piezo1-knockout osteoblasts, resolving whether TMEM63A constitutes an additional, non-redundant Ca^2+^ entry route would refine mechanistic models of osteoblast mechanobiology and help optimize non-pharmacologic bone regeneration strategies such as LIPUS. The goal of the present study is to define TMEM63A's function in osteoblast differentiation and mechanotransduction under LIPUS.

The two central hypotheses of this study include: (1) TMEM63A is required for LIPUS-induced activation of osteogenic signaling in MC3T3-E1 cells, and (2) TMEM63A loss dampens downstream mechano-responsive pathways and reduces osteogenic outputs including proliferation, migration, and differentiation. Taken together, our study provides the first functional characterization of TMEM63A in osteoblast mechanobiology and links its activity to the Wnt/β-catenin-dependent transcriptional programs documented by in vitro transcriptomics as critical mediators of LIPUS-induced osteogenesis.

## 2 Results

1 TMEM63A knockdown validation in MC3T3 cells

**Fig. 2:**
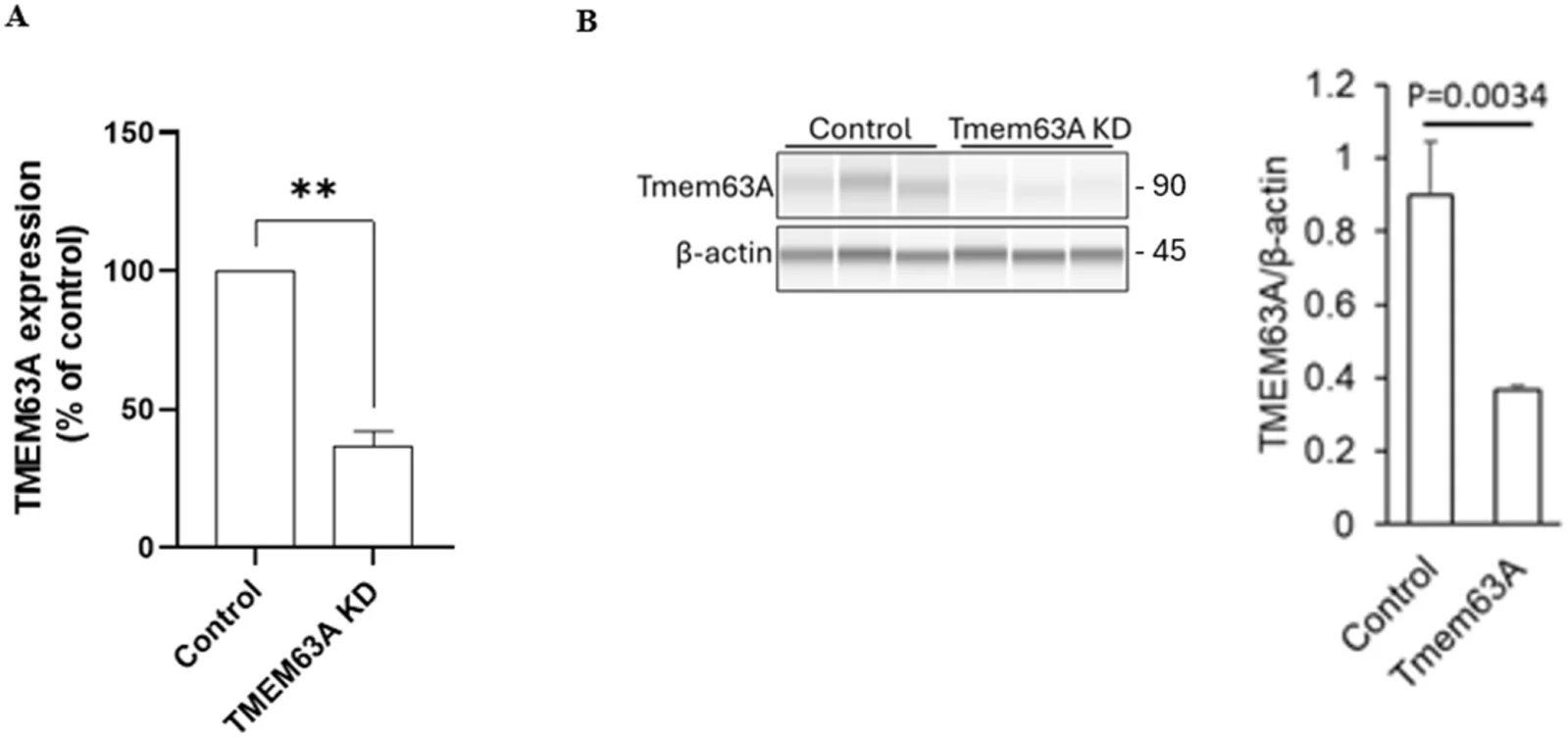
Validation of TMEM63A knockdown in MC3T3 cells. MC3T3 cells were transduced with either control or TMEM63A-specific shRNA lentiviral particles (MOI 1.5) and selected in puromycin (1.5 μg/mL) for 7 days. (A) qRT-PCR quantification of TMEM63A mRNA expression, normalized to CTR (100%). Knockdown cells show a reduction to 37 ± 4% of control. Data represent mean ± SD (n = 3); ∗∗p < 0.01 by unpaired Student's t-test. (B) Representative Western blot images for TMEM63A (∼90 kDa) and B-actin (loading control, ∼45 kDa) in control and TMEM63A knockdown cells and (C) Densitometric quantification of TMEM63A protein normalized to β-actin from three independent Western blots. Data are mean ± SD; n = 3; ∗∗p < 0.01 by unpaired two-tailed *t*-test.

We first established stable TMEM63A knockdown in MC3T3-E1 pre-osteoblasts using shRNA lentiviral transduction (MOI 1.5; 5ug/mL polybrene; 1.5ug/mL puromycin selection 7d). Quantitative RT-PCR confirmed a robust reduction of TMEM63A mRNA to 37 ± 4% of control (mean ± SD; n = 3; p < 0.01), indicating ∼63% knockdown efficiency. Western blotting corroborated the mRNA results at the protein level: TMEM63A immunoreactivity (∼90 kDa) was markedly reduced in the knockdown group, while β-actin levels remained constant. Together, these data validate the model for downstream functional assays (Fig. 2A–B).

2 TMEM63A knockdown blunts osteogenic differentiation and the pro-osteogenic effect of LIPUS

The LIPUS signal was administered using the following parameters: 30 mW/cm^2^ spatial-average temporal-average intensity, 100 Hz pulse repetition frequency, 20% duty cycle, 1 MHz frequency, and 20 min per session. The results of ALP staining to assess impact on LIPUS induced osteogenic differentiation are as follows: Without LIPUS (osteogenic induction only), at Day 7, control cells reached 43.04 ± 25.4 (x10^5^ μm^2^) ALP-positive area, whereas TMEM63A knockdown reached 15.67 ± 2.45 (x10^5^ μm^2^). By Day 14, control increased to 68.37 ± 10.34 (x10^5^ μm^2^), while knockdown remained low at 21.8 ± 4.78 (x10^5^ μm^2^), demonstrating a sustained defect in ALP induction upon TMEM63A depletion.

**Fig. 3:**
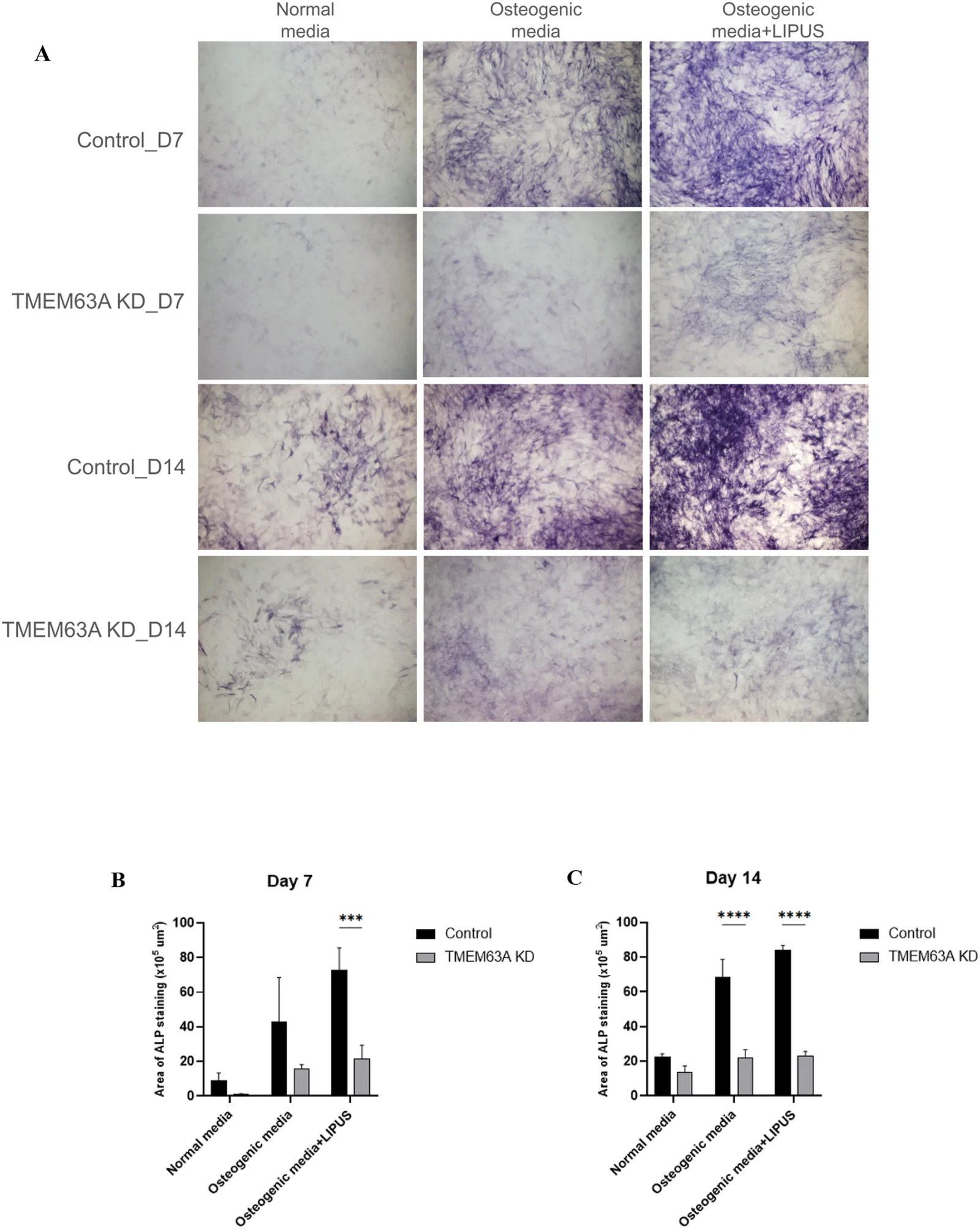
(A) Representative BCIP/NBT ALP staining of control and TMEM63A KD MC3T3-E1 cells after 7 or 14 days under (i) Normal growth medium, (ii) osteogenic induction medium, or (iii) osteogenic medium with daily LIPUS. Scale bar = 200 μm. (B) Bar graphs of normalized ALP-positive area in control and TMEM63A-shRNA cells at Days 7 and 14 of osteogenic induction with or without LIPUS (mean ± SD, n = 3; two-way ANOVA with Tukey post hoc test: ns, not significant; ∗∗∗p < 0.001; ∗∗∗∗p < 0.0001).

With daily LIPUS (20 min/day during induction, LIPUS further increased ALP area in control wells to 72.84 ± 12.72 (x10^5^ μm^2^) at Day 7 and 85.23 ± 2.59 (x10^5^ μm^2^) at Day 14. In TMEM63A-shRNA cultures, LIPUS had no significant effect (21.7 ± 7.71 (x10^5^ μm^2^) Day 7; 22.94 ± 2.64 (x10^5^ μm^2^) Day 14), indicating that TMEM63A is essential for the pro-osteogenic action of LIPUS (Fig. 3A–C).

**Fig. 4:**
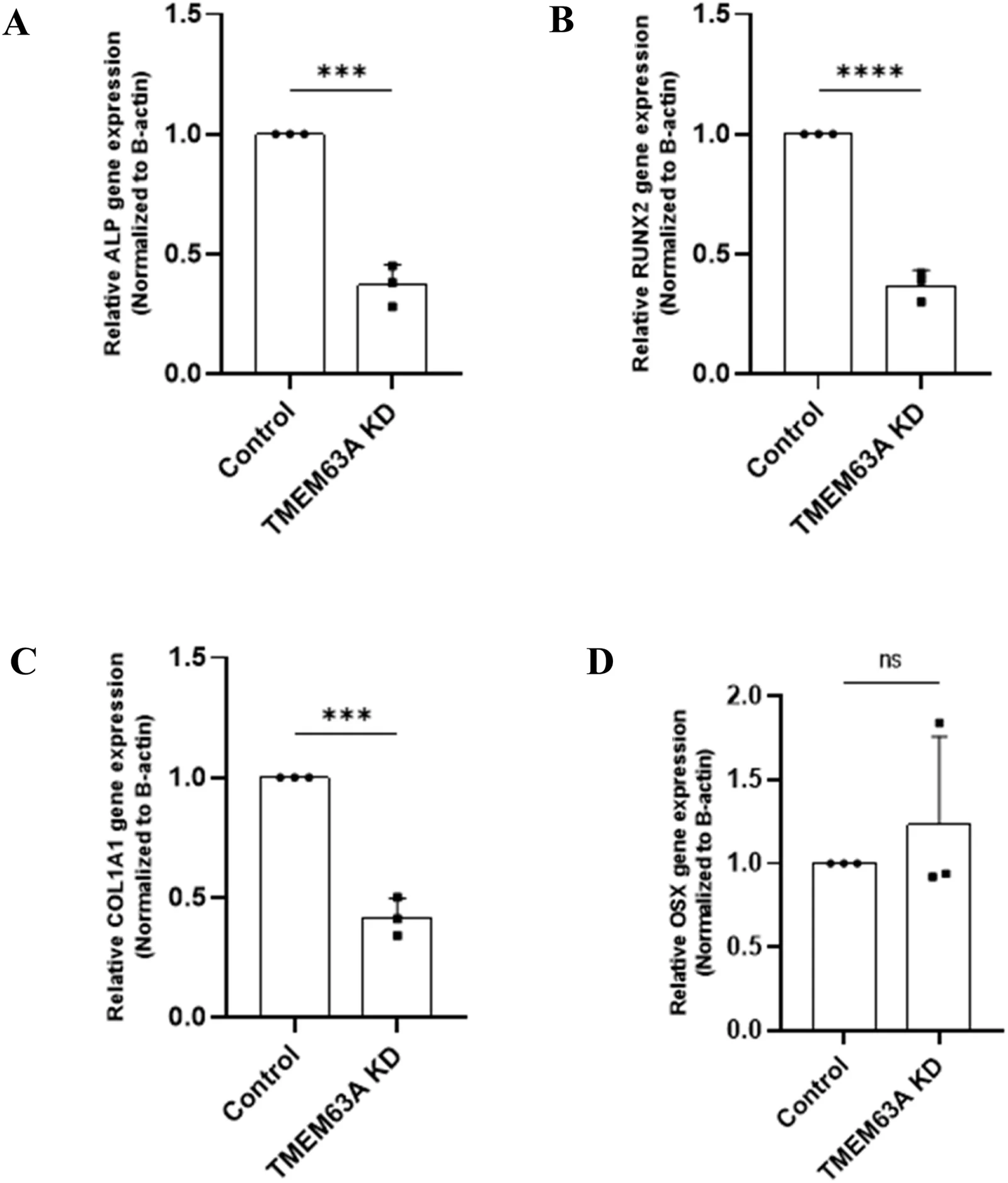
Relative mRNA expression of (A) Runx2, (B) Alp, (C) Col1a1 and (D) Osx in control versus TMEM63A KD at Day 7 of differentiation without LIPUS, normalized to β-actin and control (mean ± SD, n = 3; unpaired two-tailed *t*-test, ns = not significant; ∗∗∗p < 0.001; ∗∗∗∗p < 0.0001).

Osteogenic gene expression: TMEM63A knockdown significantly reduced the expression of multiple early osteogenic genes, with *Alp* decreasing to 0.37 ± 0.085-fold of control, *Runx2* to 0.37 ± 0.06-fold, and *Col1a1* to 0.42 ± 0.08-fold. In contrast, *Osx* expression was not significantly altered. The control and TMEM63A KD groups were maintained in osteogenic induction media alone for 7 days before extracting mRNA. (Fig. 4A–D).

3 Proliferation and migration are reduced by the loss of TMEM63A

**Fig. 5:**
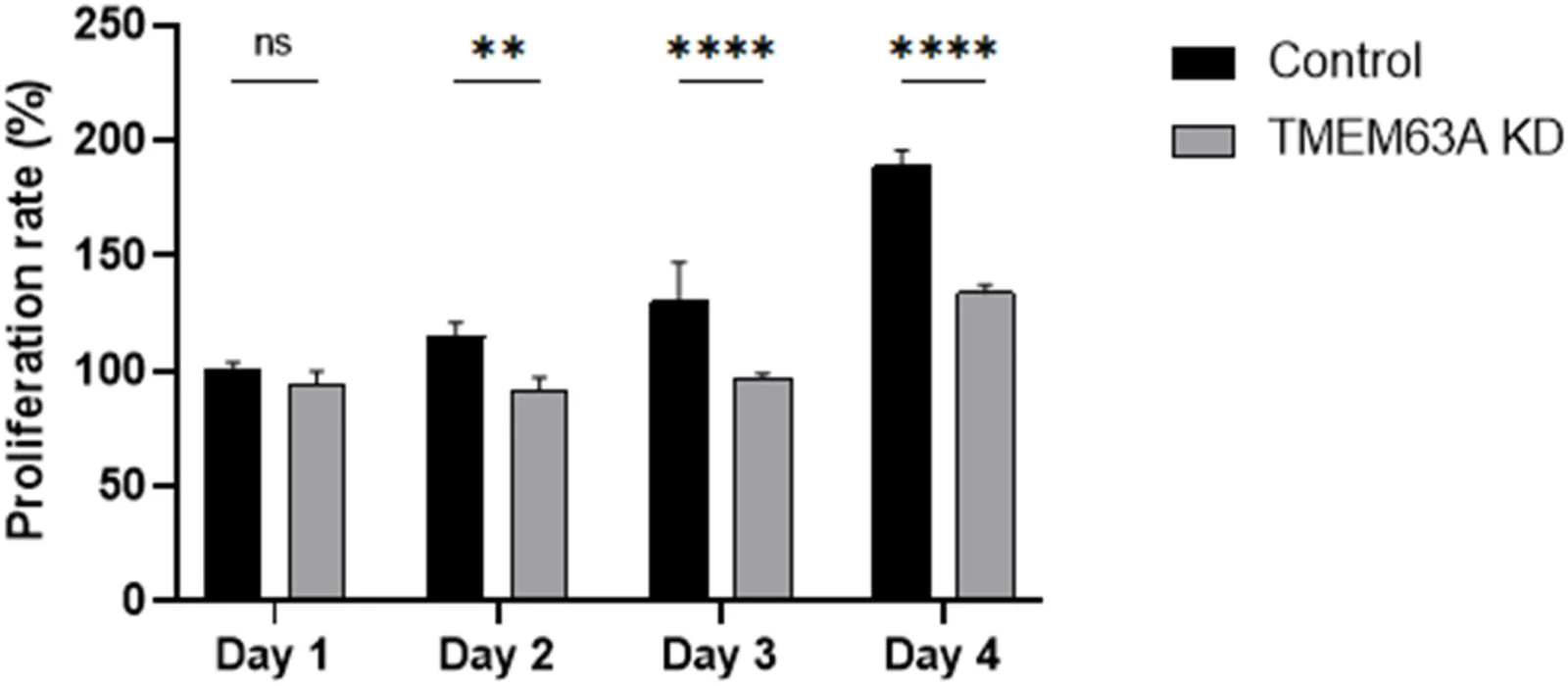
Effect of TMEM63A knockdown on MC3T3 proliferation. Cell proliferation was assessed by MTS assay on Days 1-4 post-seeding, with absorbance at 490 nm normalized to Day 1 control (100%). (mean ± SD, n = 4; two-way ANOVA: ns = not significant; ∗∗p < 0.01; ∗∗∗∗p < 0.0001).

TMEM63A knockdown did not significantly affect proliferation at Day 1 (94.4 ± 5.4% vs. 100 ± 3.6%; p > 0.05), but showed a progressive deficit by Day 2 (91.6 ± 5.6% vs. 114.9 ± 6%, p < 0.01), Day 3 (96.6 ± 2.3% vs. 130 ± 17%, p < 0.0001), and Day 4 (133 ± 3.5% vs. 188.7 ± 7.2%, p < 0.0001), indicating impaired growth over time (Fig. 5).

**Fig. 6:**
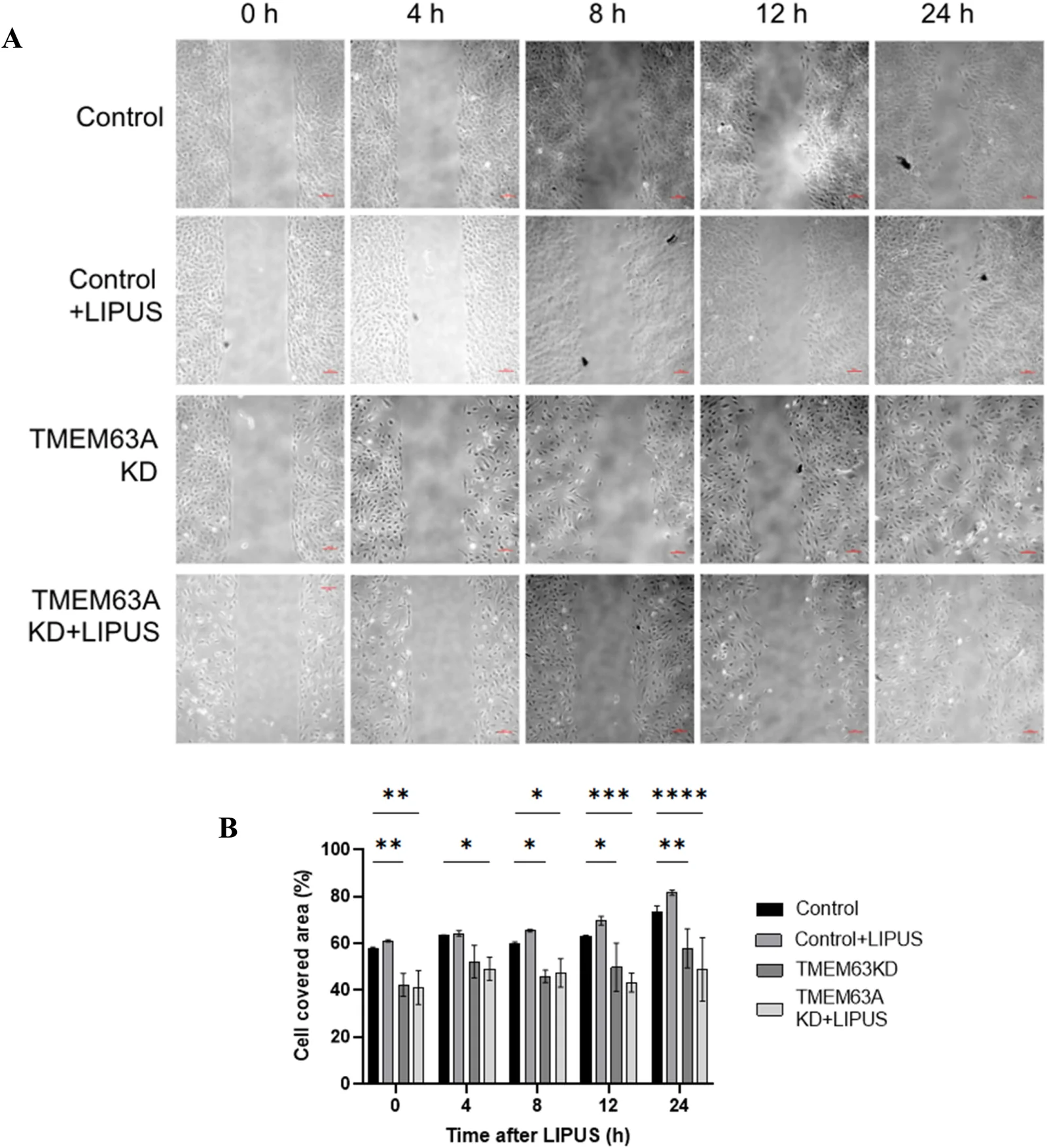
(A) Representative phase-contrast images of scratch assays at 0-24 h for control, control + LIPUS, TMEM63A KD, and TMEM63A KD + LIPUS groups (scale bar = 100 μm). (B) Quantification of percentage cell covered area over time. (C) Scratch width at each timepoint, expressed as a fraction of the width of the same sample at 0 h. (D) Migration velocity, calculated as the change in scratch width divided by twice the elapsed time to account for closure from both wound fronts. Width was measured at 5 evenly spaced positions per field, with two fields averaged per sample. (mean ± SD, n = 3; two-way ANOVA: ∗p < 0.05; ∗∗p < 0.01; ∗∗∗p < 0.001; ∗∗∗∗p < 0.0001).

To assess whether TMEM63A regulates osteoblast motility and whether LIPUS-enhanced migration depends on TMEM63A, we performed scratch wound assays in control and TMEM63A KD MC3T3-E1 cells, with and without LIPUS. Phase-contrast images were acquired at 0, 4, 8, 12, and 24 h post-wounding (Fig. 6A). We first quantified cell covered area within the standardized region of interest. In control cells, cell covered area increased from 57.9 ± 0.5% at baseline to 73.6 ± 2.4% at 24 h, and LIPUS raised this to 81.6 ± 1.1%. In TMEM63A KD cells, cell covered area reached 57.8 ± 8.4% at 24 h without LIPUS and 48.9 ± 13.6% with LIPUS (Fig. 6B). This metric, however, is not directly comparable across groups, because baseline cell covered area differed between conditions and any subsequent value therefore reflects both migration into the gap and the starting density of the monolayer. Cell covered area also cannot separate migration from proliferation, and the two groups differ in proliferative capacity (Fig. 5). We therefore repeated the analysis using scratch width, which is independent of baseline coverage, and treat that analysis as the primary readout of migration.

To assess migration independently of initial cell coverage, scratch width was quantified at 5 evenly spaced positions per field and each sample normalized to its own width at 0 h. Starting widths were comparable across groups, ranging from 779.3 ± 6.8 μm in TMEM63A knockdown to 816.0 ± 2.7 μm in TMEM63A knockdown LIPUS. LIPUS accelerated wound closure in control cells at every timepoint examined. Normalized scratch width in control LIPUS versus control was 0.842 ± 0.011 versus 0.909 ± 0.007 at 4 h, 0.699 ± 0.013 versus 0.800 ± 0.013 at 8 h, 0.609 ± 0.010 versus 0.711 ± 0.013 at 12 h, and 0.124 ± 0.005 versus 0.293 ± 0.013 at 24 h. Migration velocity showed the same pattern, rising from 9.05 ± 0.61 to 15.84 ± 1.15 μm/h at 4 h and from 11.68 ± 0.14 to 14.60 ± 0.05 μm/h at 24 h. This response was absent in TMEM63A knockdown cells. Normalized width in knockdown LIPUS versus knockdown was 0.872 ± 0.015 versus 0.921 ± 0.026 at 4 h, 0.725 ± 0.016 versus 0.695 ± 0.021 at 8 h, 0.706 ± 0.017 versus 0.709 ± 0.102 at 12 h, and 0.362 ± 0.023 versus 0.315 ± 0.075 at 24 h, with no significant difference at any timepoint. Migration velocity was likewise unchanged by LIPUS in knockdown cells at every timepoint. The LIPUS increment in velocity was 6.79, 5.18, 3.50 and 2.93 μm/h in control cells at 4, 8, 12 and 24 h, against 5.34, −0.88, 0.50 and −0.25 μm/h in knockdown cells (Fig. 6C–D). Notably, TMEM63A knockdown did not alter closure in the absence of ultrasound: control and knockdown did not differ at any timepoint for either metric. TMEM63A is therefore required for the LIPUS induced acceleration of migration but is dispensable for migration under static culture conditions. Because a proliferation deficit would be expected to slow closure under both unstimulated and stimulated conditions, the selective loss of the LIPUS response is not readily explained by the reduced proliferation reported in Fig. 5.

4 TMEM63A is required for robust LIPUS-evoked calcium dynamics in MC3T3 cells

**Fig. 7:**
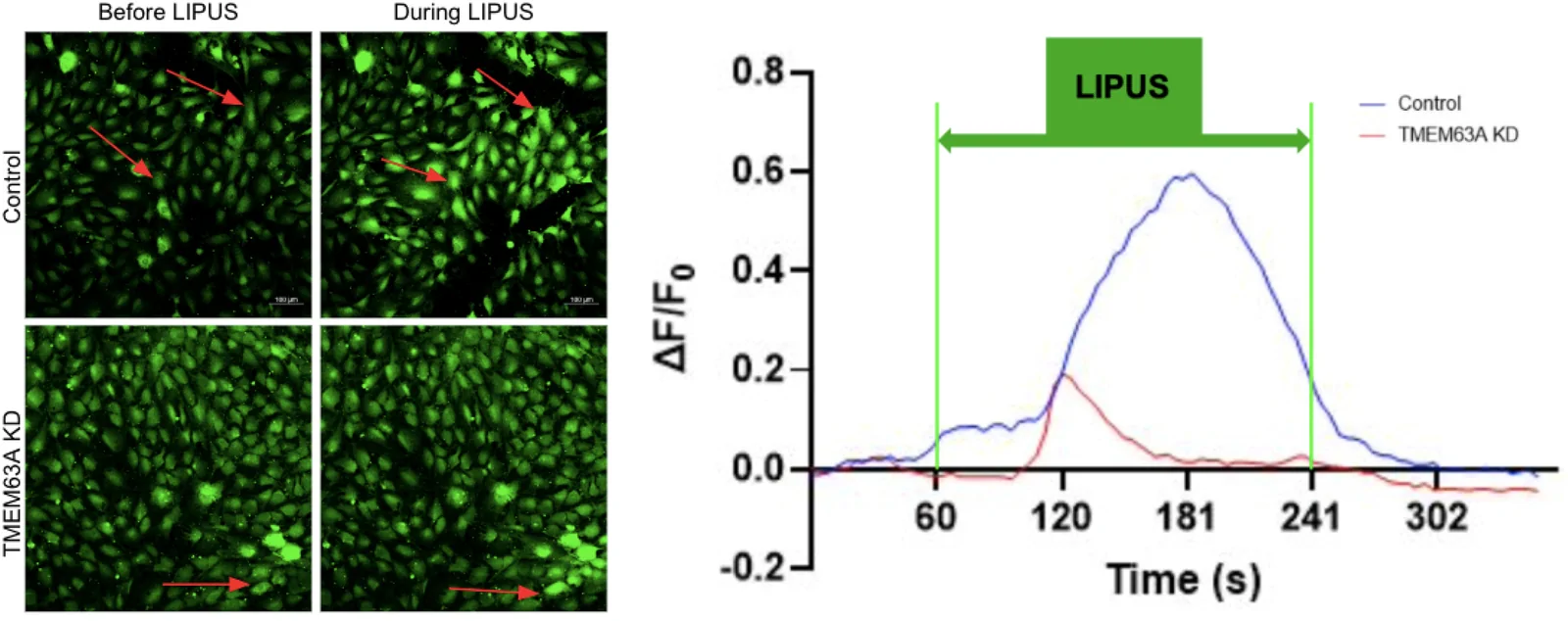
(Left) Representative Fluo-4 AM live cell images of intracellular Ca^2+^ in scrambled control and TMEM63A knockdown (KD) MC3T3-E1 cells, captured at baseline before stimulation and during active LIPUS stimulation. Increased Fluo-4 green fluorescence reflects elevated cytosolic Ca^2+^. Red arrows indicate responding cells in which fluorescence rises during LIPUS. Scale bar = 100 μm. (Right) LIPUS-evoked Ca^2+^ dynamics expressed as ΔF/F_0_ over time, where ΔF/F_0_ = (F − F_0_)/F_0_ and F_0_ is the mean baseline fluorescence before stimulation, in scrambled control (blue) and TMEM63A KD (red) cells. Peak ΔF/F_0_ reaches approximately 0.60 in control cells versus approximately 0.18 to 0.20 in KD cells, a reduction of approximately 65 to 70%. Traces represent means, n = 3 independent experiments with at least 10 cells per experiment.

To test whether TMEM63A functions upstream of mechanotransduction through Ca^2+^ signaling, we performed live-cell calcium imaging during/after acute stimulation. In control MC3T3 cells, LIPUS elicited a prominent calcium response characterized by a rapid rise followed by a broad peak and decay [Fig. 7 (right)]. The control trace reached a peak ΔF/F_0_ ≈ 0.60, occurring at approximately ∼180 s, and remained elevated for ∼1-2 min before returning toward baseline.

In contrast, TMEM63A knockdown (KD) cells exhibited a markedly attenuated response. KD cells showed a smaller, brief elevation with a peak ΔF/F_0_ ≈ 0.18-0.20 near ∼120 s, followed by a faster decay back toward baseline [Fig. 7 (right)]. Relative to control, TMEM63A KD reduced peak amplitude by ∼65-70% (0.20 vs 0.60) and shortened the response duration, consistent with impaired mechanically induced Ca^2+^ entry.

5 LIPUS promotes β-catenin nuclear translocation in control cells but not after TMEM63A knockdown

Because Wnt/β-catenin is a major osteogenic pathway, we next tested whether LIPUS drives β-catenin nuclear accumulation and whether this requires TMEM63A. β-catenin localization was quantified as a nuclear/cytoplasmic (N/C) intensity ratio.

**Fig. 8:**
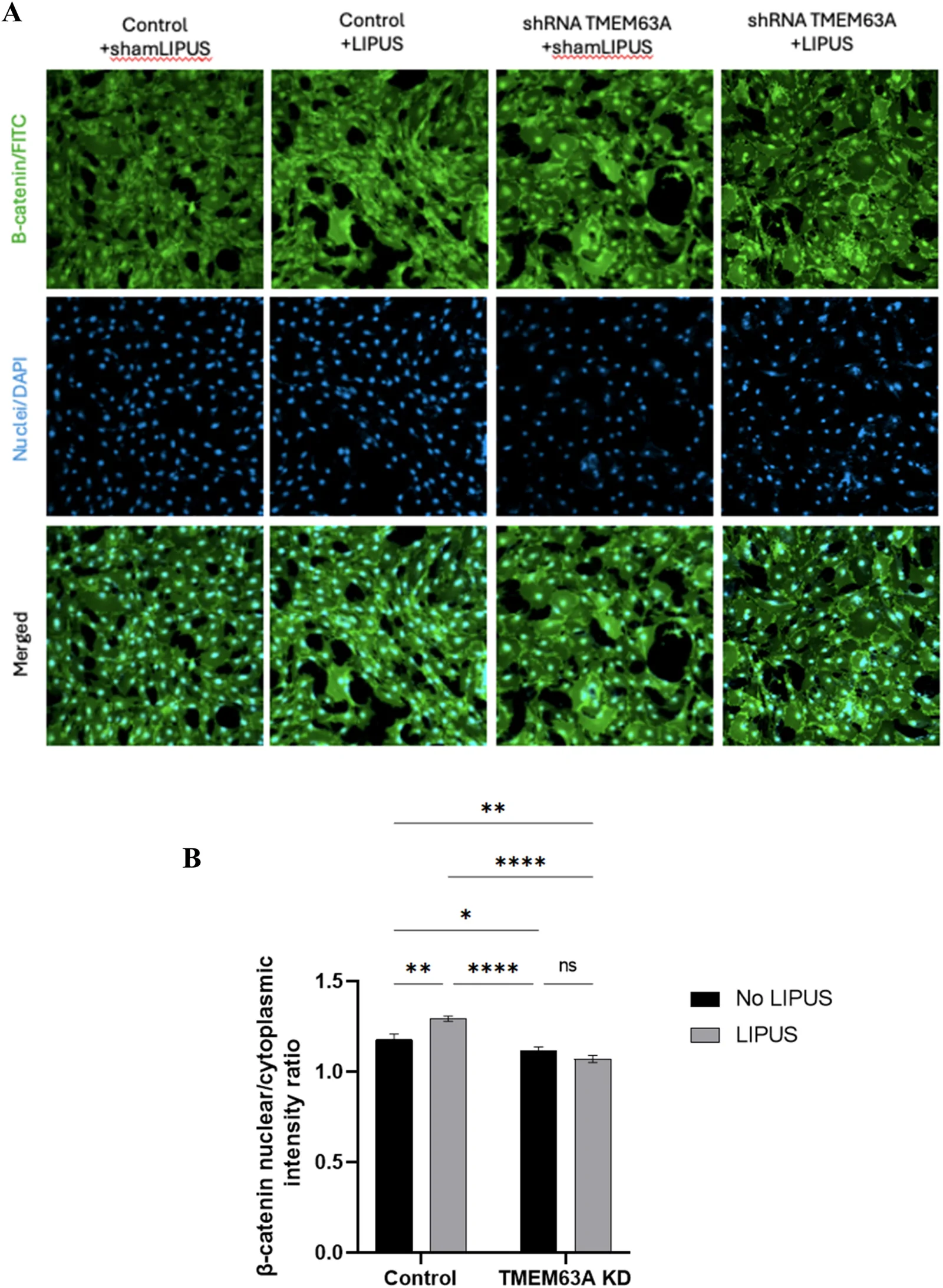
(A) Representative images of β-catenin nuclear translocation in (1) shRNA-control MC3T3-E1 cells treated without LIPUS stimulation, (2) shRNA-control MC3T3-E1 cells treated with LIPUS stimulation, (3) shRNA-TMEM63A MC3T3-E1 cells treated without LIPUS stimulation, (4) shRNA-TMEM63A MC3T3-E1 cells treated with LIPUS stimulation. (B) β-catenin Nuclear/Cytoplasmic intensity ratio. Control vs LIPUS shows ∼13% increase (p < 0.01). TMEM63A KD vs KD + LIPUS shows no significant difference (ns). Data are mean ± SD. Statistical analysis was by two-way ANOVA: ns, not significant; ∗p < 0.05, ∗∗p < 0.01, ∗∗∗∗p < 0.0001.

In control cells, LIPUS significantly increased β-catenin N/C ratio (Fig. 8B), rising from approximately ∼1.15 (Control) to ∼1.30 (LIPUS). This indicates that LIPUS enhances β-catenin nuclear enrichment under conditions where TMEM63A is intact.

In TMEM63A KD cells, β-catenin N/C ratios were not significantly altered by LIPUS (Fig. 8B). The KD baseline was approximately ∼1.10, and the LIPUS condition remained near ∼1.05 (ns). Thus, TMEM63A depletion eliminated the LIPUS-induced β-catenin nuclear shift observed in controls.

6 Genome-wide transcriptomic profiling reveals coordinated disruption of osteogenic, ECM, and PI3K/AKT/Wnt programs in TMEM63A knockdown cells

**Fig. 9:**
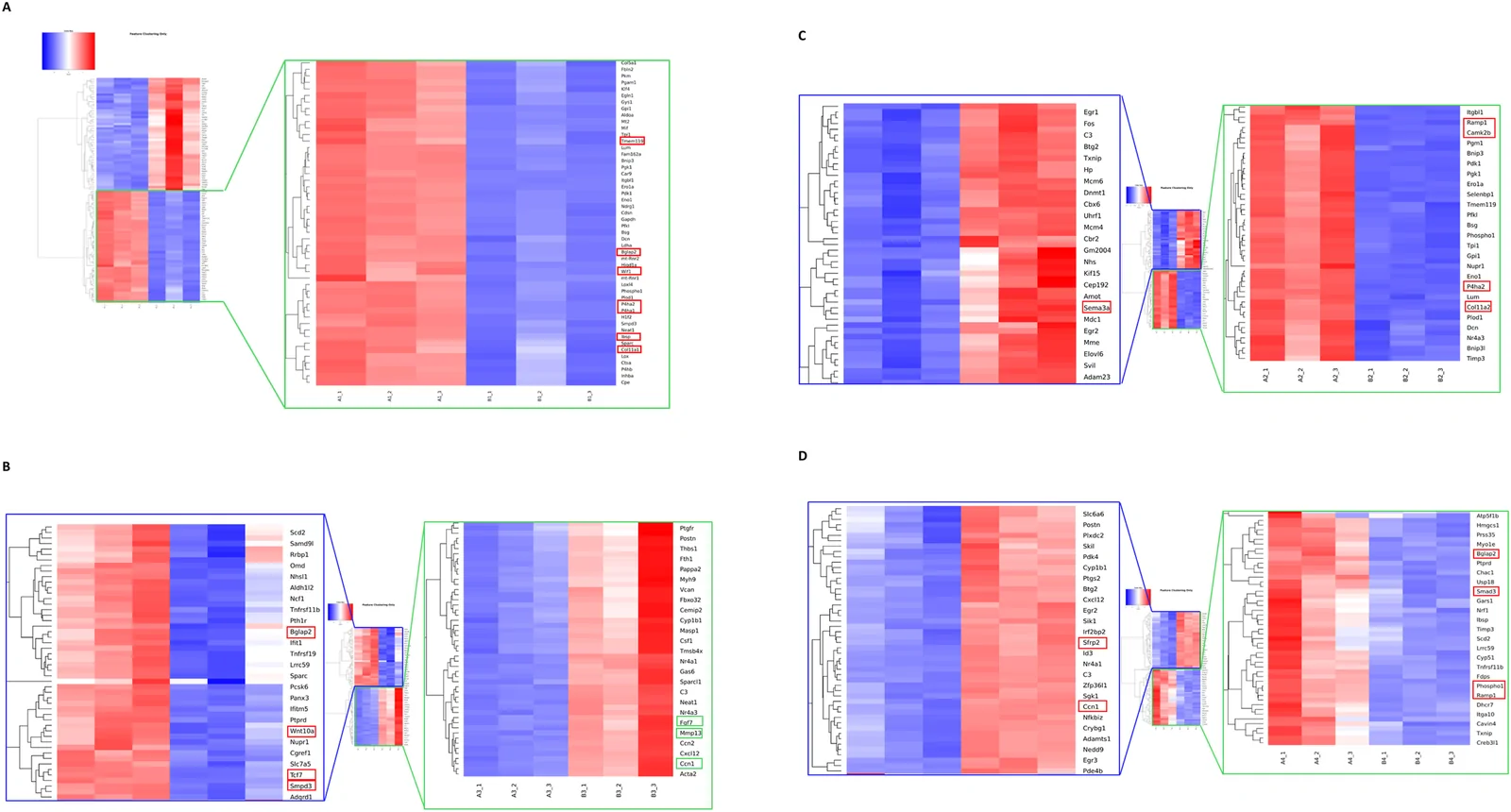
Hierarchical clustering heatmaps of top 100 differentially expressed genes for four comparisons. Control (A1_1, A1_2, A1_3) vs. TMEM63A-KD (B1_1, B1_2, B1_3) at Day 7 (A), Control (A3_1, A3_2, A3_3) vs. TMEM63A-KD (B3_1, B3_2, B3_3) at Day 14 (B), Control + LIPUS (A2_1, A2_2, A2_3) vs. TMEM63A-KD + LIPUS (B2_1, B2_2, B2_3) at Day 7 (C), and Control + LIPUS (A4_1, A4_2, A4_3) vs. TMEM63A-KD + LIPUS (B4_1, B4_2, B4_3) at Day 14 (D). Rows represent individual DEGs, and columns represent biological replicates (n = 3 per condition). Color scale represents row-scaled z-scores (red = high relative expression, blue = low). Clear separation between control and knockdown samples is evident across all four comparisons, indicating that the TMEM63A-loss transcriptional phenotype is robust and persists under LIPUS stimulation and selected genes are highlighted in red boxes.

To define the global transcriptional consequences of TMEM63A loss during osteogenic differentiation and to test whether LIPUS-induced gene programs require TMEM63A, we performed bulk RNA sequencing on control and TMEM63A-shRNA MC3T3-E1 cells at Day 7 and Day 14 of osteogenic induction, with or without daily LIPUS exposure (n = 3 per condition; 12 samples total). Hierarchical clustering of differentially expressed genes confirmed clear separation of control and knockdown samples across all four conditions (Fig. 9, heatmaps), indicating a robust transcriptional phenotype that persists in the presence of LIPUS.

**Fig. 10:**
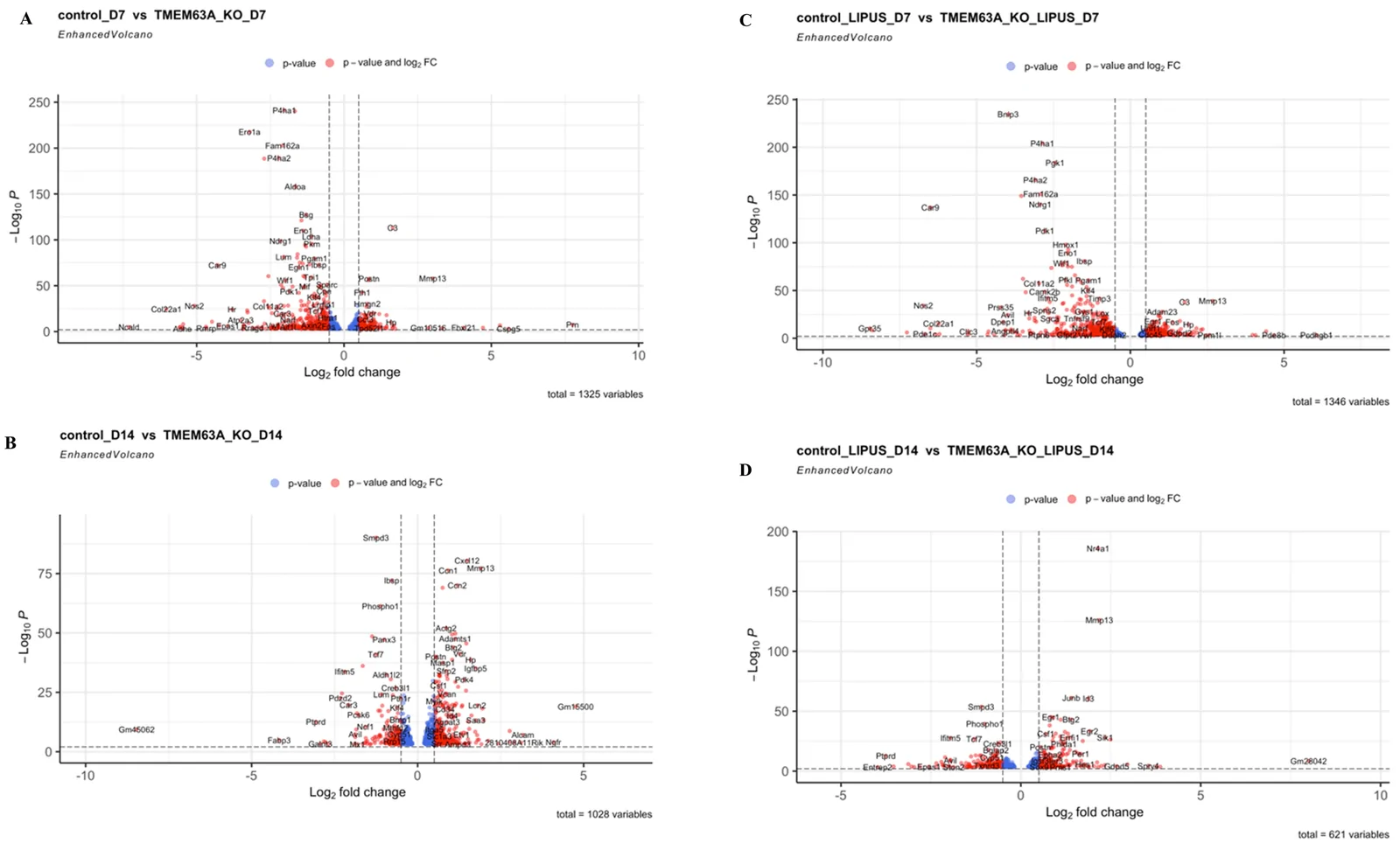
Volcano plots of differentially expressed genes across the four pairwise comparisons. Control vs. TMEM63A-KD at Day 7 (A), Day 14 (B), Day 7 + LIPUS (C), and Day 14 + LIPUS (D). Red dots represent genes meeting both significance and fold-change thresholds (padj ≤ 0.05 and |log_2_FC| ≥ 0.5); blue dots represent genes meeting only the p-value threshold. Selected top DEGs are labeled.

In Fig. 9, collagen processing and crosslinking enzymes (P4ha1, P4ha2), fibrillar collagens (Col11a2, Col22a1), and bone matrix proteins (Bsp/Ibsp, Sparc, Pcolce) were among the most significantly downregulated transcripts in TMEM63A-knockdown cells at Day 7, indicating compromised collagen biosynthesis and matrix deposition. Among the downregulated transcripts in groups with LIPUS treatment was Camk2b, a Ca2+/calmodulin-dependent kinase that is directly downstream of channel-mediated Ca2+ signaling and offers a direct transcriptional correlate of the compromised Ca2+ dynamics shown in our imaging experiments (Fig. 7), in addition to Wnt effectors Tcf7, Wif1, and Axin2. On the other hand, knockdown cells showed an increase in the matrix metalloproteinases Mmp13, suggesting a shift from matrix deposition toward matrix degradation, as shown in Fig. 10.

**Fig. 11:**
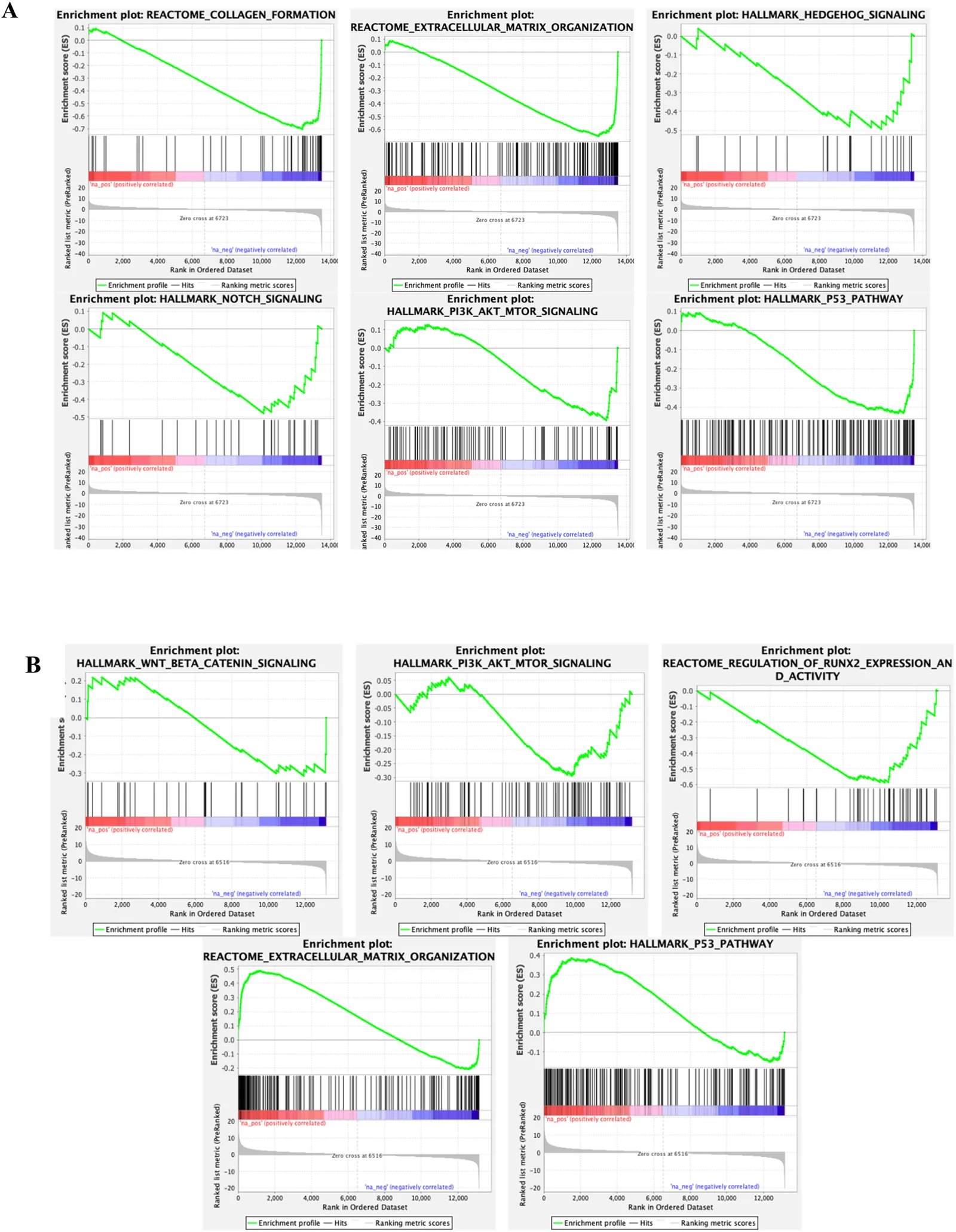
Selected GSEA enrichment plots for Day 7 and Day 14 control vs. TMEM63A-KD comparisons (no LIPUS). (A) Negative enrichment of Reactome collagen formation, ECM organization, was observed at Day 7, alongside Hallmark PI3K-AKT-mTOR signaling, P53 signaling, Hedgehog signaling, and Notch signaling. (B) At Day 14, negative enrichment of Hallmark Wnt/β-catenin signaling, Hallmark PI3K-AKT-mTOR signaling, and Reactome regulation of RUNX2 expression and activity was observed, alongside positive enrichment of Hallmark P53 pathway and Reactome ECM organization. Each plot displays the running enrichment score (green line) and the position of gene set members in the ranked gene list (vertical bars).

Gene set enrichment analysis (GSEA) of the Hallmark and Reactome gene sets revealed a transcriptional phenotype that evolved temporally. At Day 7, TMEM63A-knockdown cells exhibited coordinated negative enrichment across matrix and adhesion programs, including Reactome collagen formation, ECM organization, indicating widespread suppression of both matrix synthesis and matrix turnover machinery. Negative enrichment was also seen for Hallmark PI3K-AKT-mTOR signaling, P53 signaling, Hedgehog signaling, and Notch signaling, indicating a widespread early failure to engage anabolic and developmental signaling systems (Fig. 11A). On Day 14, the transcriptional phenotype divided into two opposing arms. The osteogenic and anabolic pathways were suppressed, resulting in negative enrichment of Hallmark Wnt/β-catenin signaling, Hallmark PI3K-AKT-mTOR signaling, and Reactome control of RUNX2 expression and activity. In contrast, knockdown cells showed a positive enrichment in matrix-degradation and remodeling programs, such as Reactome ECM organization and Hallmark P53 signaling (Fig. 11B). Thus, by Day 14, TMEM63A deletion is associated with a coordinated shift away from Wnt- and AKT-driven osteogenic anabolism and toward a matrix-degrading, remodeling transcriptional state, which corresponds to the upregulation of Mmp13 found in the differential expression analysis.

**Fig. 12:**
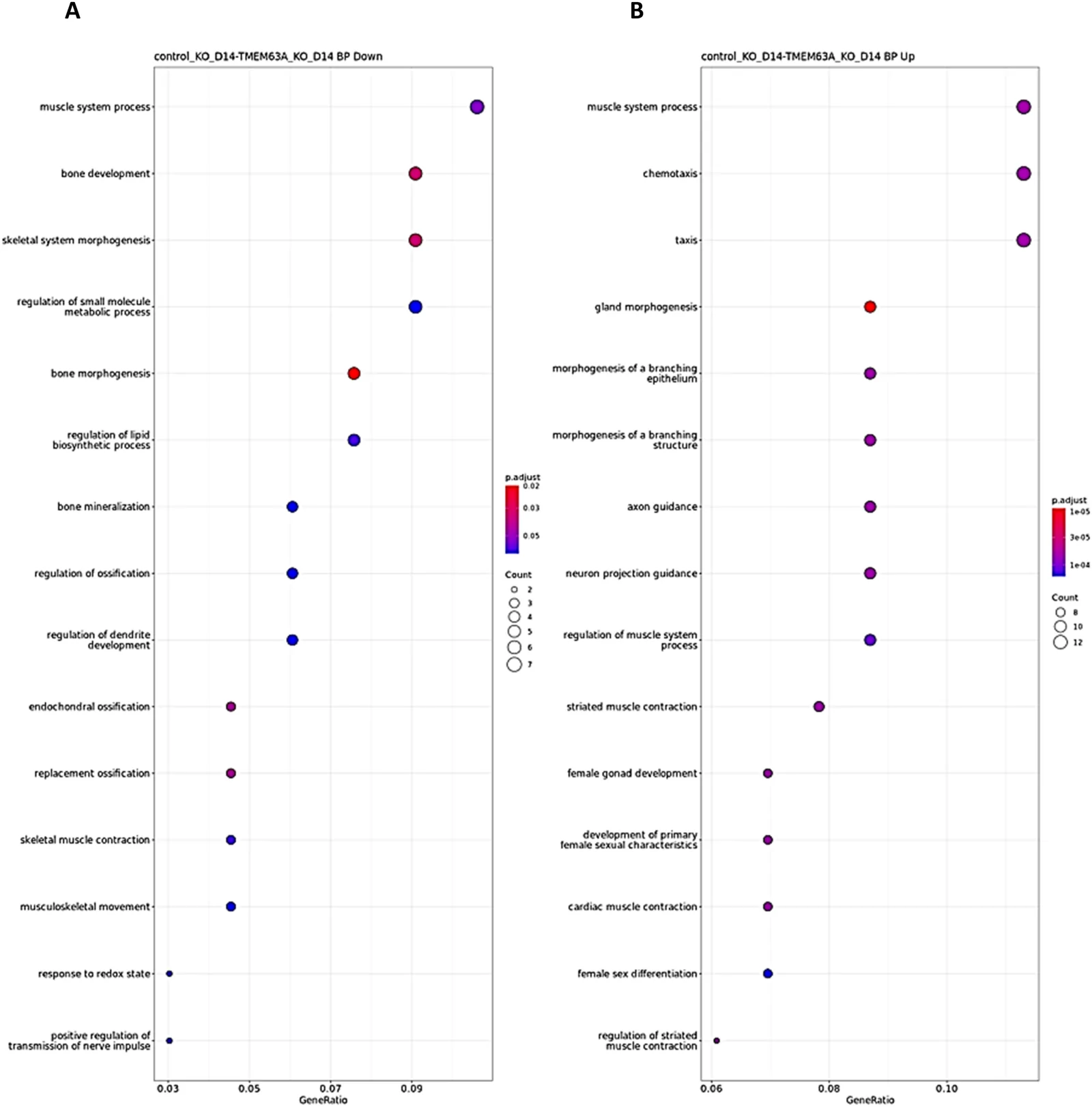
Gene Ontology biological process (BP) dot plots for downregulated (A) and upregulated (B) DEGs in TMEM63A-knockdown cells (Day 14, no LIPUS**).** Top 15 enriched GO BP terms are shown. Dot size represents gene count; color represents adjusted p-value (red = lower p-value, blue = higher); x-axis represents GeneRatio. Downregulated terms are dominated by skeletal-specific programs (bone development, skeletal system morphogenesis, bone morphogenesis, bone mineralization, regulation of ossification, endochondral ossification), demonstrating coordinated transcriptional disengagement of osteogenic programs. Upregulated terms include muscle system process, axon guidance.

This conclusion was supported by a Gene Ontology examination of biological processes (Fig. 12). At Day 14, skeletal-specific programs-bone development, skeletal system morphogenesis, bone morphogenesis, bone mineralization, regulation of ossification, endochondral ossification, replacement ossification, and biomineral tissue development-dominated the downregulated GO terms in the knockdown. These terms suggest that TMEM63A depletion interferes with the coordinated transcriptional engagement of osteogenic programs rather than just slowing differentiation.

**Fig. 13:**
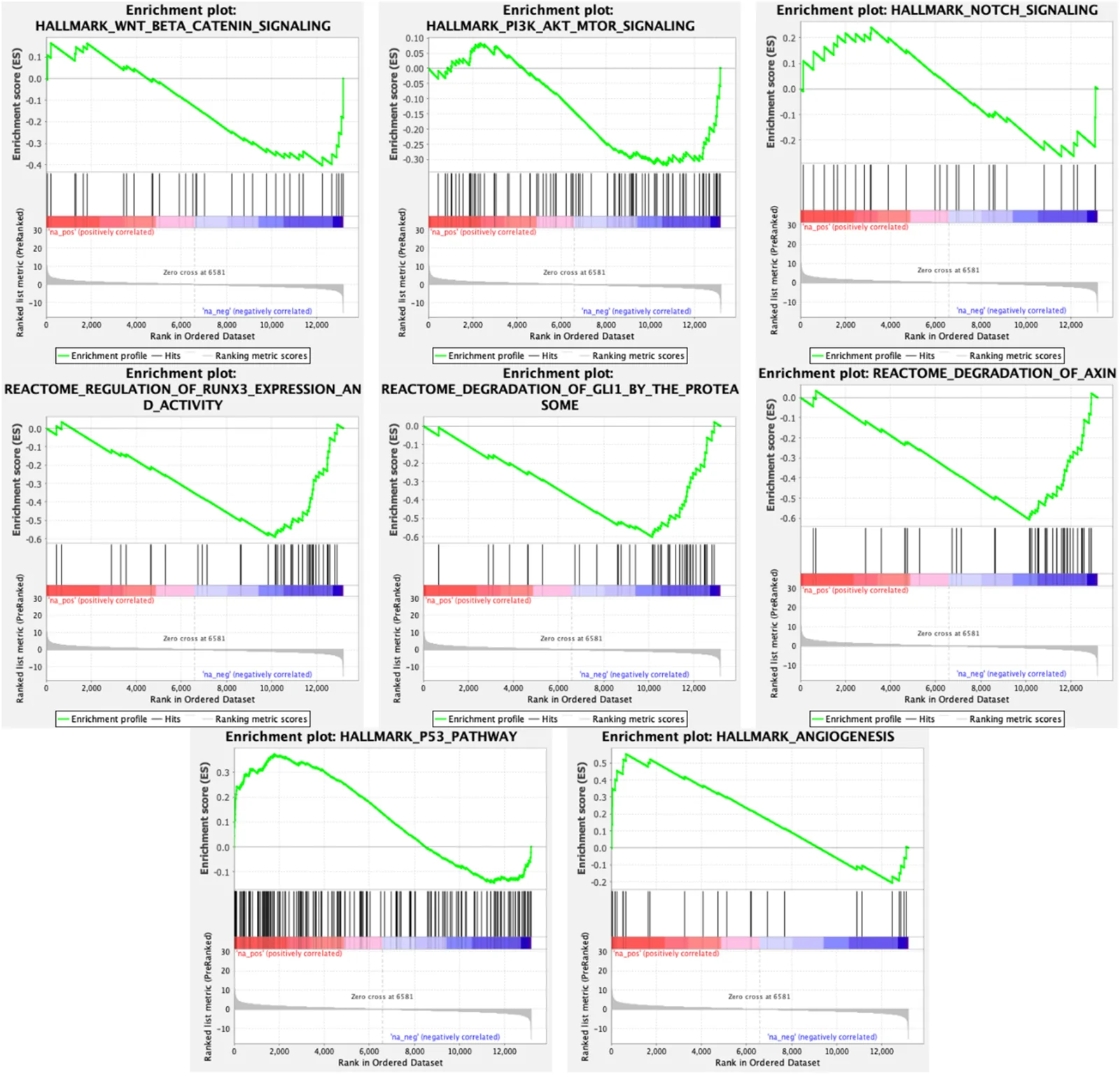
Selected GSEA enrichment plots for the Day 14 control + LIPUS vs. TMEM63A-KD + LIPUS comparison. Negative enrichment of Hallmark Wnt/β-catenin signaling, Hallmark PI3K-AKT-mTOR signaling, Hallmark Notch signaling is shown alongside Reactome regulation of RUNX3 expression and activity, Reactome degradation of Axin, and Reactome degradation of Gli1.

**Fig. 14:**
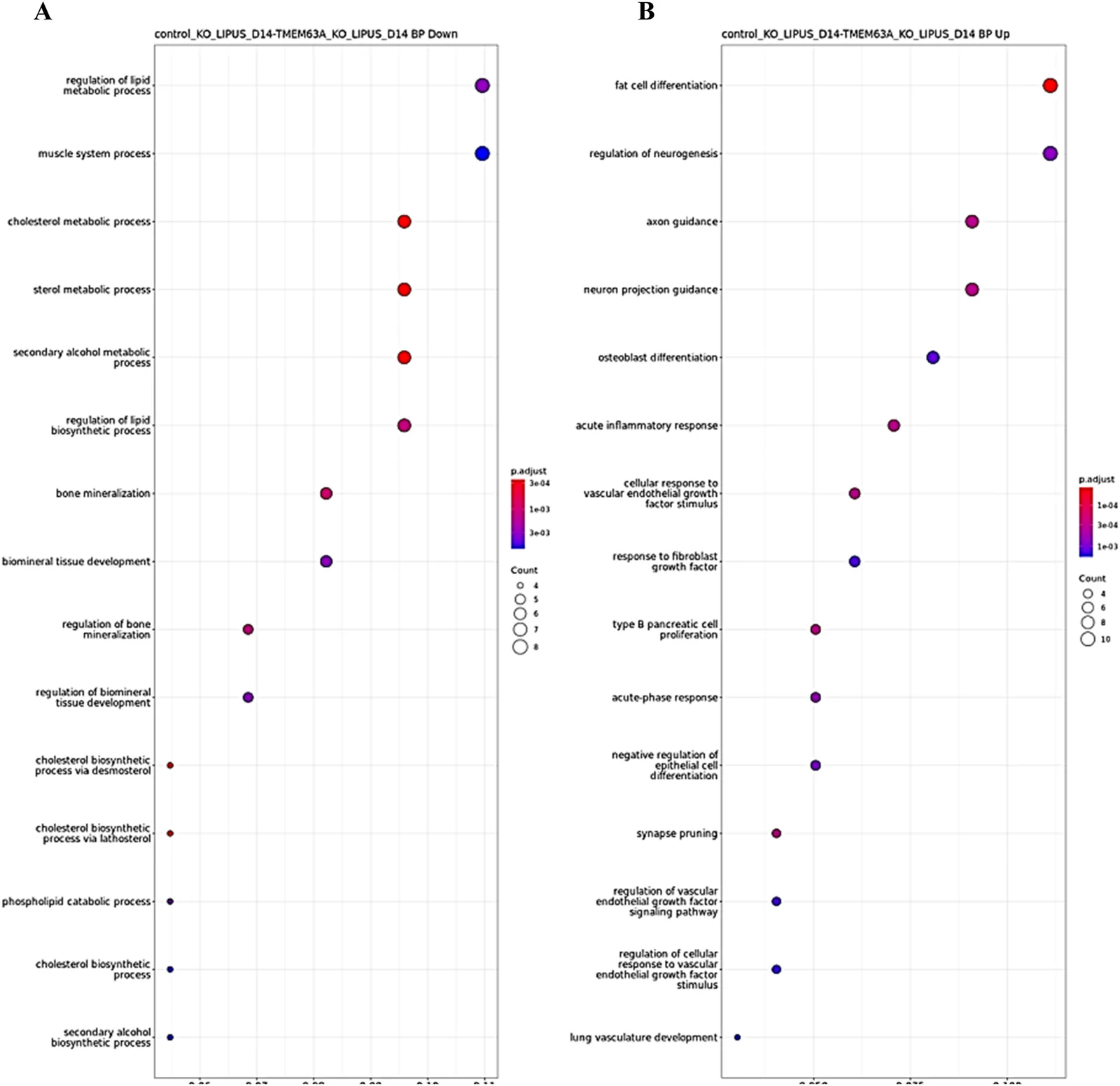
Gene Ontology biological process dot plots for downregulated and upregulated DEGs in TMEM63A-knockdown cells under LIPUS stimulation (Day 14). Downregulated (A) GO BP terms include, bone mineralization, regulation of bone mineralization, and regulation of biomineral tissue development, mirroring the no-LIPUS condition and indicating that LIPUS does not rescue osteogenic transcriptional programs in the absence of TMEM63A. Upregulated (B) terms include fat cell differentiation, regulation of neurogenesis, axon guidance, neuron projection guidance, and osteoblast differentiation.

We then investigated if LIPUS could repair the transcriptional deficiency in knockdown cells. At Day 14, GSEA showed negative enrichment of Hallmark Wnt/β-catenin signaling, Hallmark PI3K-AKT-mTOR signaling, Hallmark Notch signaling, Hallmark P53 signaling, Reactome regulation of RUNX3 expression and activity, and Reactome collagen degradation (Fig. 13). LIPUS-treated knockdown cells showed negative enrichment for Reactome degradation of Axin and Gli1. This suggests that the destruction complexes targeting both β-catenin (via Axin scaffolding) and Gli1 (Hedgehog effector) remain transcriptionally active in TMEM63A-deficient cells under LIPUS stimulation. The absence of Axin destruction in LIPUS-treated knockdown cells suggests that the Wnt/β-catenin destruction complex is still active and targets β-catenin for proteasomal degradation. This is consistent with the observed reduced nuclear β-catenin in our imaging experiments (Fig. 8). The gene ontology analysis of the LIPUS-treated Day 14 comparison revealed sustained downregulation of bone mineralization, regulation of bone mineralization, and biomineral tissue development in knockdown cells (Fig. 14). These studies show that the loss of TMEM63A prevents the activation of LIPUS-induced osteogenic gene programs. Additionally, the failure to alleviate GSK3β-mediated β-catenin degradation explains the problem.

7 LIPUS-induced AKT phosphorylation requires TMEM63A

**Fig. 15:**
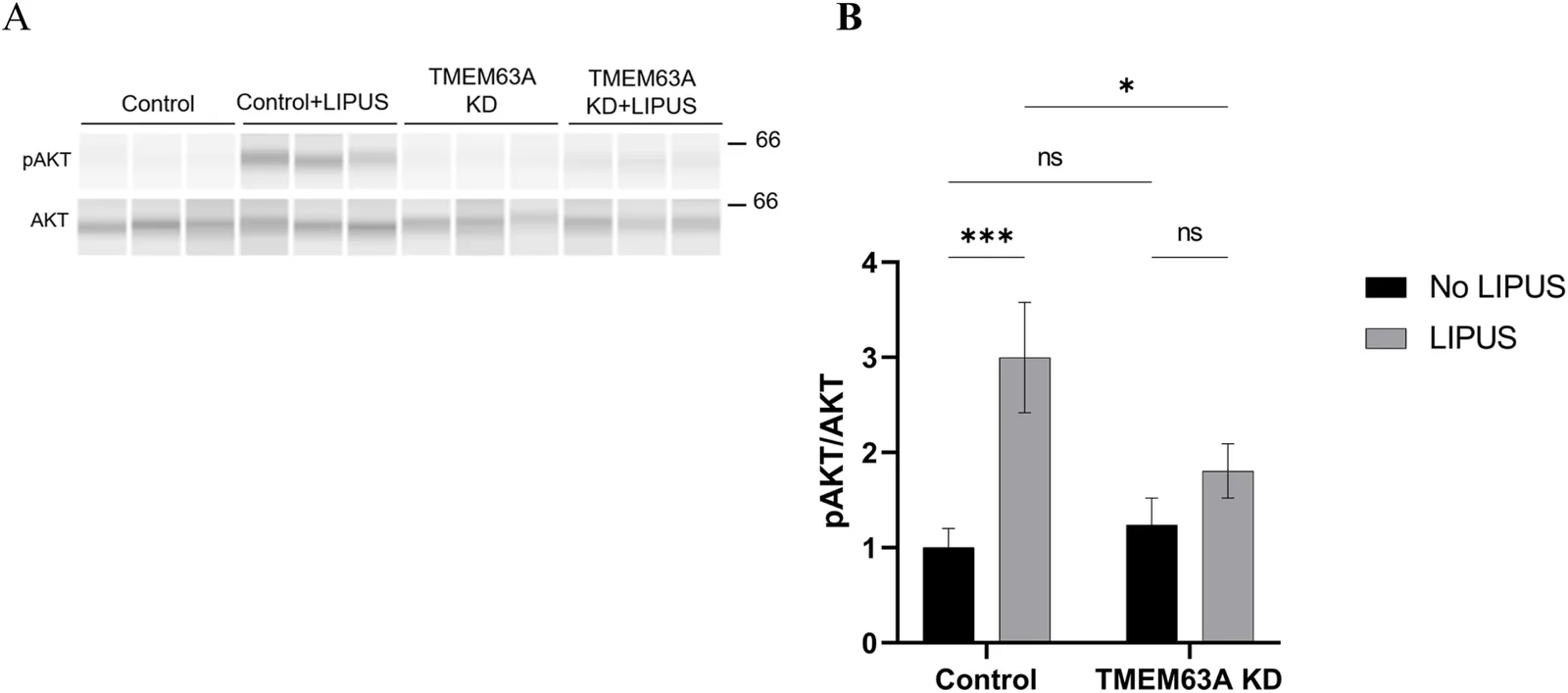
(A) Representative immunoblots for phosphorylated AKT (pAKT, ∼66 kDa) and total AKT (∼66 kDa) in control and TMEM63A-knockdown (KD) MC3T3-E1 cells, each without or with a LIPUS. LIPUS increased pAKT in control cells but not in TMEM63A-knockdown cells, whereas total AKT was comparable across all conditions. Apparent molecular weight is indicated at right (66 kDa). (B) Densitometric quantification of pAKT normalized to total AKT, expressed as fold change relative to untreated control (mean ± SD; n = 3; two-way ANOVA; ns = not significant; ∗p < 0.05; ∗∗∗p < 0.001).

To determine if TMEM63A is necessary for LIPUS-induced engagement of the PI3K/AKT node, we performed immunoblotting in control and TMEM63A-knockdown MC3T3-E1 cells, both with and without LIPUS after 14 days of differentiation. In control cells, LIPUS significantly increased phosphorylated AKT (pAKT) compared to unstimulated controls, whereas total AKT remained unaltered. In TMEM63A-knockdown cells, basal pAKT was low, and LIPUS did not raise it significantly, while total AKT remained consistent across all circumstances. LIPUS-induced AKT phosphorylation needs TMEM63A, confirming the AKT node predicted by RNA sequencing and placing it upstream of GSK3β inhibition and β-catenin stabilization in the suggested process (Fig. 15).

## 3 Discussion

**Fig. 16:**
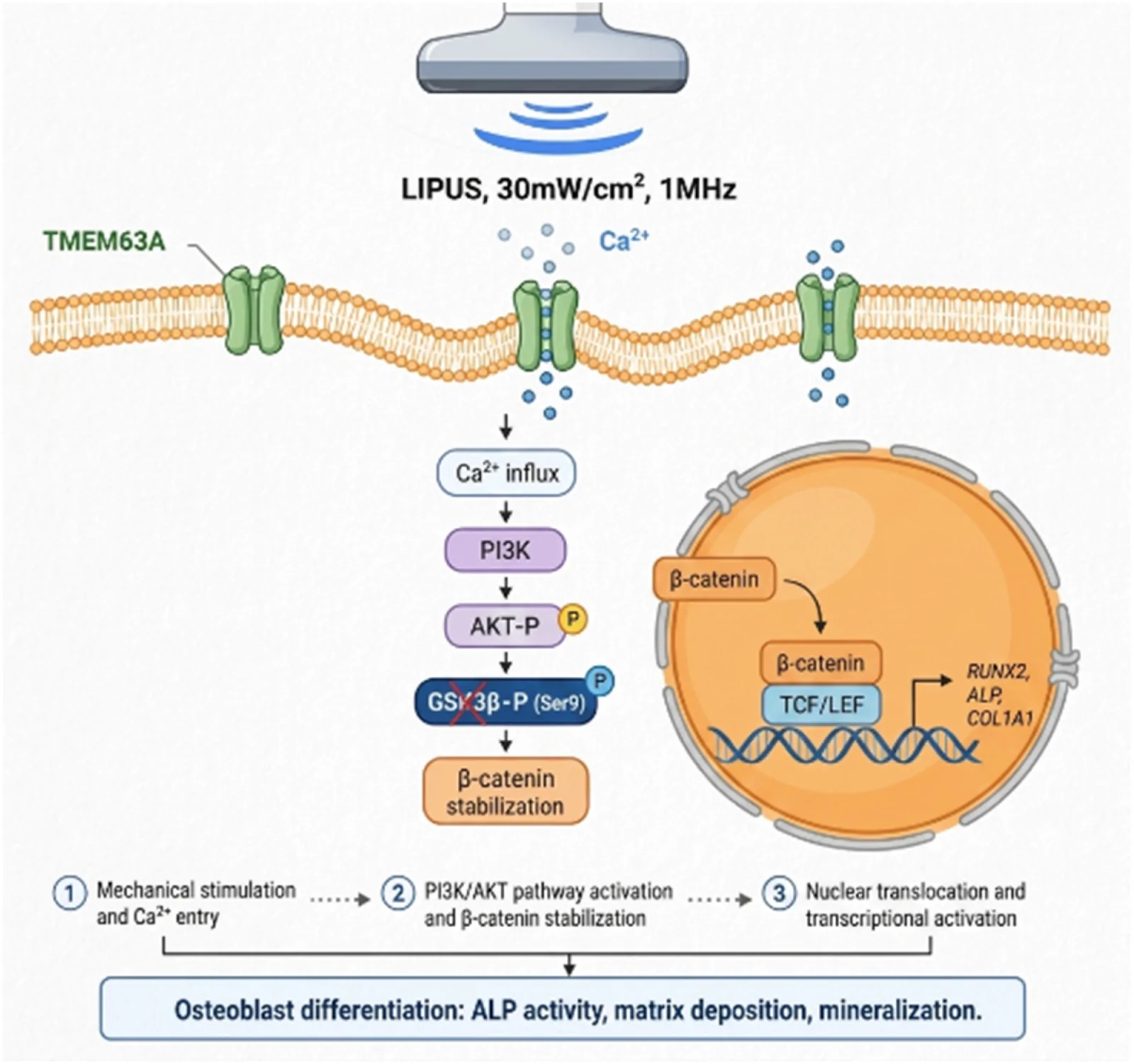
**Proposed mechanism of TMEM63A function in LIPUS-induced osteoblast differentiation.** Schematic depicting the inferred mechanism by which TMEM63A couples LIPUS-induced membrane deformation to Wnt/β-catenin activation and osteogenic transcription. Acoustic radiation force from LIPUS produces nanoscale membrane deformation that activates the TMEM63A cation channel. TMEM63A-mediated Ca^2+^ influx engages PI3K/AKT signaling, leading to Ser9 phosphorylation and inhibition of GSK3β. Inhibited GSK3β can no longer phosphorylate β-catenin within the cytoplasmic destruction complex, allowing β-catenin to escape proteasomal degradation, accumulate in the cytoplasm, and translocate to the nucleus. Nuclear β-catenin associates with TCF/LEF transcription factors to drive expression of *Runx2*, *Alp*, and *Col1a1*, ultimately promoting alkaline phosphatase activity, matrix deposition, and mineralization. The model integrates findings from this study including calcium imaging, β-catenin nuclear translocation, and transcriptomic profiling, including the genome-wide negative enrichment of Reactome degradation of Axin under LIPUS in TMEM63A-knockdown cells. Phosphorylation of AKT (Ser473) was confirmed by Western blot and phosphorylation of GSK3β (Ser9) is predicted by transcriptomic data and remains to be confirmed at the protein level. Created in https://BioRender.com.

In this study, we identified TMEM63A as a necessary mechanosensor that links low-intensity pulsed ultrasound to osteogenic differentiation in pre-osteoblasts. Stable shRNA-mediated suppression of TMEM63A in MC3T3-E1 cells decreased alkaline phosphatase activity, proliferation, migration, and the expression of the early osteogenic transcription factors *Runx2*, *Alp*, and *Col1a1*. Knockdown cells showed significantly reduced LIPUS-induced Ca^2+^ entry and nuclear translocation of β-catenin. Genome-wide RNA sequencing revealed that TMEM63A knockdown impairs not just individual osteogenic genes, but also the coordinated transcriptional engagement of bone formation, mineralization, and ossification programs, with LIPUS not able to rescue these deficiencies. Our findings suggest that TMEM63A acts as a proximal mechanosensor, connecting LIPUS to the Ca^2+^ → AKT → GSK3β → β-catenin axis that drives osteogenic transcription (Fig. 16).

TMEM63A's biophysical properties, namely a high activation threshold, slow gating kinetics, and monomeric architecture,^31^^,^^32^ distinguish it from rapidly inactivating, low threshold channels such as PIEZO1. The extent of TMEM63A inactivation is not consistent across the literature. Murthy et al.^32^ report that mouse TMEM63A inactivates with a time constant of 323 ms, approximately eleven fold slower than the 28.8 ms reported for mouse PIEZO1 under equivalent stretch conditions, while describing human TMEM63A currents as largely non-inactivating, with an exponential fit obtainable in only two of nine recordings. Zheng et al.^31^ and Niloy et al.^33^ report that TMEM63A currents lack inactivation altogether, plateauing within 2 s and remaining sustained for at least 5 s. These studies differ in stimulus duration, construct, and recording configuration, and the discrepancy is unresolved. What is consistent across all of them is that inactivation, where it occurs, is at least an order of magnitude slower than PIEZO1, and that activation is correspondingly slow, with a time constant of 126 ms for mouse TMEM63A against 9.0 ms for PIEZO1. These kinetics have a specific consequence for our stimulus. An ultrasound stimulator (Sonicator 740) was used to deliver 2 ms bursts at a pulse repetition frequency of 100 Hz, separated by 8 ms intervals. This period falls between the gating timescales of the two channel families. PIEZO1, activating with a time constant of ∼9.0 ms and inactivating with a time constant of ∼28.8 ms, operates on a timescale comparable to the stimulus period and would be expected to track and progressively filter the pulse train, consistent with the frequency dependent transduction^35^ described for repetitive mechanical stimulation. TMEM63A, activating with a time constant of ∼126 ms and inactivating no faster than approximately 323 ms, is one to two orders of magnitude slower than the stimulus period. It contributes little conductance within any single 2 ms burst but loses correspondingly little during the 8 ms interval, and therefore integrates across the approximately 120,000 bursts delivered in a 20-min exposure, responding to the time-averaged membrane tension rather than to the pulse structure of the stimulus. Niloy et al.^33^ found, using paired pressure steps across a range of inter-pulse intervals, that TMEM63A currents recover quickly between successive stimuli, indicating that the channel does not become refractory under repetitive activation. TMEM63A governs Ca^2+^ entry during each exposure; persistence arises downstream, through AKT phosphorylation, GSK3β inhibition, β-catenin stabilization and nuclear translocation, and osteogenic transcription, reinforced by daily repeated exposure across the differentiation window. TMEM63A is the proximal transducer at each exposure rather than the element that stores the response.

In patch clamp experiments with mechanical indentation as the stimulus, the peak mechanically activated current dropped from 150 picoamps in wild-type cells to about 66 in the Piezo1 KO cells. 43% of the currents still persisted without PIEZO1. That residual current was attributed to the presence of other mechanosensors and also residual PIEZO1 protein.^20^ TMEM63A is also shown to be mechanically regulated: proteomics from human osteoblasts under simulated microgravity showed TMEM63A is among the top ten downregulated proteins, when mechanical load was reduced, TMEM63A synthesis was reduced, as expected of a load-sensing gene.^34^ We showed that TMEM63A knockdown cells exhibit a ∼65% reduction in LIPUS-induced Ca^2+^ oscillations, suggesting that TMEM63A plays a significant role in cation entry. This magnitude of reduction indicates that PIEZO1 does not compensate for the loss of TMEM63A under our stimulation conditions, although we have not tested the two channels in combination and cannot exclude compensation under different mechanical regimes. The persistence of mechanically activated current in Piezo1 knockout osteoblasts^20^ is consistent with a PIEZO independent component of osteoblast mechanotransduction, and the more severe skeletal phenotype of Piezo1/2 double knockout relative to either single knockout^22^ illustrates that mechanosensory contributions in this lineage are distributed across multiple channels. The migration data locate TMEM63A within the mechanically evoked arm of the osteoblast response rather than within its baseline motility. Knockdown left wound closure unaltered under static conditions at every timepoint, while abolishing the acceleration produced by LIPUS, and the genotype by stimulus interaction was significant for both scratch width and migration velocity. This is the pattern expected of a mechanotransducer rather than of a general regulator of cytoskeletal function, and it parallels the reduction in LIPUS evoked Ca^2+^ oscillations reported above.

Our genome-wide transcriptomic analysis provides corroboration of the AKT-GSK3β-β-catenin axis, and reveals a temporally evolving phenotype. At Day 7, TMEM63A loss broadly suppressed the matrix and adhesion machinery, with negative enrichment of Reactome collagen formation, ECM organization, and collagen and ECM degradation, alongside Hallmark PI3K-AKT-mTOR, P53, Hedgehog, and Notch signaling. By Day 14, the phenotype had diverged into two opposing arms. The osteogenic and anabolic programs remained suppressed, with negative enrichment of Hallmark Wnt/β-catenin signaling, Hallmark PI3K-AKT-mTOR signaling, and Reactome regulation of RUNX2 and RUNX3 expression. In contrast, matrix-degradation and remodeling programs were positively enriched in knockdown cells, including Reactome collagen degradation, ECM degradation, ECM organization, and cell-extracellular matrix interactions, alongside Hallmark P53 signaling. This divergence is consistent with the reciprocal upregulation of *Mmp13* observed in the differential expression analysis and indicates that, in the absence of TMEM63A, osteoblasts shift away from a matrix-depositing, Wnt-active anabolic state toward a matrix-degrading, remodeling state. Under LIPUS at Day 14, this same divergent pattern persisted: Hallmark Wnt/β-catenin, Hallmark PI3K-AKT-mTOR, Reactome regulation of RUNX3 expression, and Reactome degradation of Axin all remained negatively enriched in knockdown cells, while matrix-degradation programs remained positively enriched. Because degradation of Axin is a positive readout of active canonical Wnt signaling, its suppression in LIPUS-treated knockdown cells provides genome-wide evidence that the β-catenin destruction complex remains assembled and continues to target β-catenin for degradation, paralleling the reduced nuclear β-catenin observed in our β-catenin translocation imaging experiments. The convergence of two independent transcriptional readouts, Wnt/β-catenin Hallmark suppression and Axin-degradation suppression, together with the imaging-level loss of nuclear β-catenin, establishes that TMEM63A is required to dismantle the β-catenin destruction complex in response to mechanical stimulation, and that LIPUS alone cannot bypass this requirement. Our findings parallel the LIPUS-driven FAK → PI3K → AKT → GSK3β → β-catenin cascade recently reported in BMSCs.^24^ Combined with the coordinated reduction of bone-specific GO biological processes (bone development, mineralization, ossification, and biomineral tissue development), these findings position TMEM63A as a required upstream component of the integrated AKT-β-catenin-RUNX2 axis driving osteoblast differentiation.

The therapeutic efficacy of LIPUS for fracture healing has been inconsistent among numerous investigations. The TRUST randomized controlled trial of operatively managed tibial fractures discovered no improvement in radiographic healing or functional recovery with postoperative LIPUS,^29^ and a subsequent BMJ Rapid Recommendations systematic review concluded that LIPUS does not accelerate return to work, return to full weight bearing, or pain reduction across available randomized evidence.^30^ Our findings suggest a possible mechanistic explanation for clinical heterogeneity: if patient-to-patient variation in TMEM63A expression, function, or genetic background determines whether the AKT-GSK3β-β-catenin axis can engage in response to ultrasound, the average clinical effect across an unstratified population may underestimate the benefit in TMEM63A-competent responders while overstating it in non-responders. Defining TMEM63A as a proximal mechanosensor for LIPUS suggests that its expression or polymorphism could serve as a biomarker for stratifying responders. Combining LIPUS with pharmacological GSK3β inhibition may bypass the channel requirement in non-responders. To determine whether such stratification can regain clinical benefit, prospective trials with mechanism-informed patient selection are required.

Several limitations of this study must be acknowledged. First, our knockdown approach used a single shRNA construct. Although a non-targeting scrambled shRNA control was included and convergent phenotypes were observed across multiple independent functional assays (alkaline phosphatase, proliferation, migration, gene expression, calcium imaging, β-catenin localization, and unbiased RNA-seq), the use of a single shRNA does not formally exclude off-target contributions to the observed phenotypes. Genetic rescue using a shRNA-resistant TMEM63A construct, and confirmation with a second independent shRNA targeting a distinct region of *Tmem63a*, will be important to definitively attribute the observed effects to TMEM63A loss. Second, our study does not investigate whether TMEM63A is essential for osteogenic induction via mechanical stimulation in the absence of chemical induction. Because all groups received osteogenic medium, the results show that TMEM63A is required for the LIPUS response in an osteogenically committed context, but they cannot tell whether TMEM63A mediates a mechanically driven osteogenic program independent of chemical induction or amplifies a program initiated by the medium. Reports that mechanical stimulation alone can increase osteogenic marker expression make this distinction worth addressing. A LIPUS arm in growth medium, with and without TMEM63A knockdown, would distinguish between the two possibilities. Our exposure parameters differ from the convention most commonly used in the LIPUS bone healing literature, which employs a 1.5 MHz carrier with 200 μs bursts at a 1 kHz pulse repetition frequency. The ultrasound stimulator (Sonicator 740) delivers 2 ms bursts at 100 Hz, so both burst duration and inter-burst interval are approximately ten-fold longer, and the carrier frequency is 1 MHz. This device has been used previously for mechanotransduction studies in MC3T3-E1 cells at these settings,^2^ and the 20 percent duty cycle and low-intensity regime are shared with standard protocols, but direct quantitative comparison of our outcomes with studies using 1 kHz devices should be made with this difference in mind. Third, our investigation was limited to MC3T3-E1 cells, a clonal mouse pre-osteoblast line. Validation in primary osteoblasts, bone marrow stromal cells, and in vivo mouse models will be required to determine the role of TMEM63A throughout the osteoblast lineage and in the intact bone microenvironment. Fourth, we report transducer output rather than the acoustic field at the cell monolayer. Reflection at the medium and air interface and at the well walls, standing wave formation, and the impedance mismatch of the plate produce spatial variation in pressure amplitude that our stated intensity does not capture; Hensel et al.^36^ measured local peak pressure increases of up to five fold from these effects in culture wells, and showed that a 2.56 percent change in medium volume alters peak rarefaction pressure at the cell layer twofold. Conditions during live Ca^2+^ imaging differed further, with the transducer submerged 6 mm above a glass-bottomed dish and the cells within the applicator near field. We did not perform hydrophone mapping, so our comparisons rest on the presence or absence of a TMEM63A dependent response rather than on a quantitative dose relationship. Because proliferation was not blocked during the scratch assay, closure reflects both migration and proliferation. Formally isolating migration would require mitomycin C or an equivalent proliferation block, and we therefore interpret these data alongside the proliferation results rather than as an independent measure of motility. Our RNA-seq data suggest that the PI3K-AKT-mTOR and Wnt/β-catenin pathways are involved at the transcriptional level, and our β-catenin localization data confirms engagement at the protein level. Direct immunoblotting confirmed the LIPUS-induced phospho-AKT response predicted by these data: LIPUS increased phosphorylated AKT in control osteoblasts, whereas this induction was abolished in TMEM63A-knockdown cells despite unchanged total AKT, providing protein-level validation of the AKT node upstream of β-catenin. Confirmation of the corresponding phospho-GSK3β (Ser9) response remains to be established, and analysis of this target is ongoing.

Because TMEM63A activates and deactivates slowly in comparison to the rapidly deactivating PIEZO1,^14^^,^^32^ it may preferentially transduce sustained or repetitive loading, whereas PIEZO1 encodes transient high-frequency events; whether these kinetic differences translate into distinct contributions to long-term bone formation will require conditional deletion of Tmem63a alone or in combination with Piezo1 in osteoblast-lineage cells in vivo. Cx43-mediated coupling and paracrine ATP and PGE_2_ signaling spread mechanical information from osteocytes to osteoblasts and osteoclasts.^13^ It remains to be determined if TMEM63A acts cell-autonomously or affects multicellular load responses across the osteoblasts, osteoclasts, and osteocytes.

In conclusion, we present the first functional characterization of TMEM63A in osteoblast biology and identify it as a necessary mechanosensor coupling LIPUS-induced membrane deformation to a Ca^2+^ → AKT → β-catenin axis, promoting osteogenic transcription. Genome-wide transcriptome profiling reveals that this system is active not only at individual signaling nodes, but also at the level of coordinated osteogenic gene programs, and LIPUS cannot rescue these programs in the absence of TMEM63A. Beyond identifying a previously unknown component of the bone mechanosensory machinery, our findings give a molecular foundation that may help optimize LIPUS treatment procedures and patient classification in clinical fracture management.

## 4 Materials and methods

### 4.1 Cell culture and osteogenic induction

MC3T3-E1 subclone 4 pre-osteoblasts (ATCC CRL-2593, Manassas, VA) were grown in α-Minimum Essential Medium (α-MEM; Gibco, NY) with 10% fetal bovine serum (FBS; Gibco, NY) and 1% penicillin-streptomycin (Gibco, NY) at 37°C in a humidified environment of 5% CO_2_. Cells were passaged at 80-90% confluence with 0.5% trypsin-EDTA (Gibco, NY) and used between passages 5 and 25 in all studies. Cells were grown in osteogenic induction medium (OIM) containing α-MEM, 10% FBS, 1% penicillin-streptomycin, 50 μg/mL ascorbic acid (Sigma-Aldrich, St. Louis, MO), 10 mM β-glycerophosphate (Sigma-Aldrich, St. Louis, MO). OIM was refreshed every 2-3 days during the differentiation period. This design ensured osteogenic supplementation was held constant across groups, so the effect of TMEM63A knockdown could be attributed to the mechanical variable rather than differences in chemical induction. We did not include a LIPUS arm in growth medium without osteogenic supplements, and we address the consequences of this choice in the Limitations.

### 4.2 Lentiviral transduction and selection

MC3T3-E1 cells were seeded at a density of 50,000 cells per well in 24-well plates. To establish stable knockdown and control MC3T3-E1 lines, we employed a lentiviral shRNA construct targeting mouse Tmem63a (Santa Cruz Biotechnology, Dallas, TX) and a non-targeting scrambled shRNA control (Santa Cruz Biotechnology, Dallas, TX). MC3T3-E1 cells at 50% confluence were transduced with lentiviral particles at a multiplicity of infection of 1.5 and treated with 5 μg/mL polybrene (Santa Cruz Biotechnology, Dallas, TX) for 24 h. After 24 h of transduction, cells were selected with 1.5 μg/mL puromycin (Gibco, A1113803) for 7 days. Before being used in downstream research, surviving cells were grown and verified for knockdown efficiency at the mRNA and protein levels using qRT-PCR and Western blotting, respectively.

### 4.3 LIPUS apparatus and stimulation protocols

**Fig. 17:**
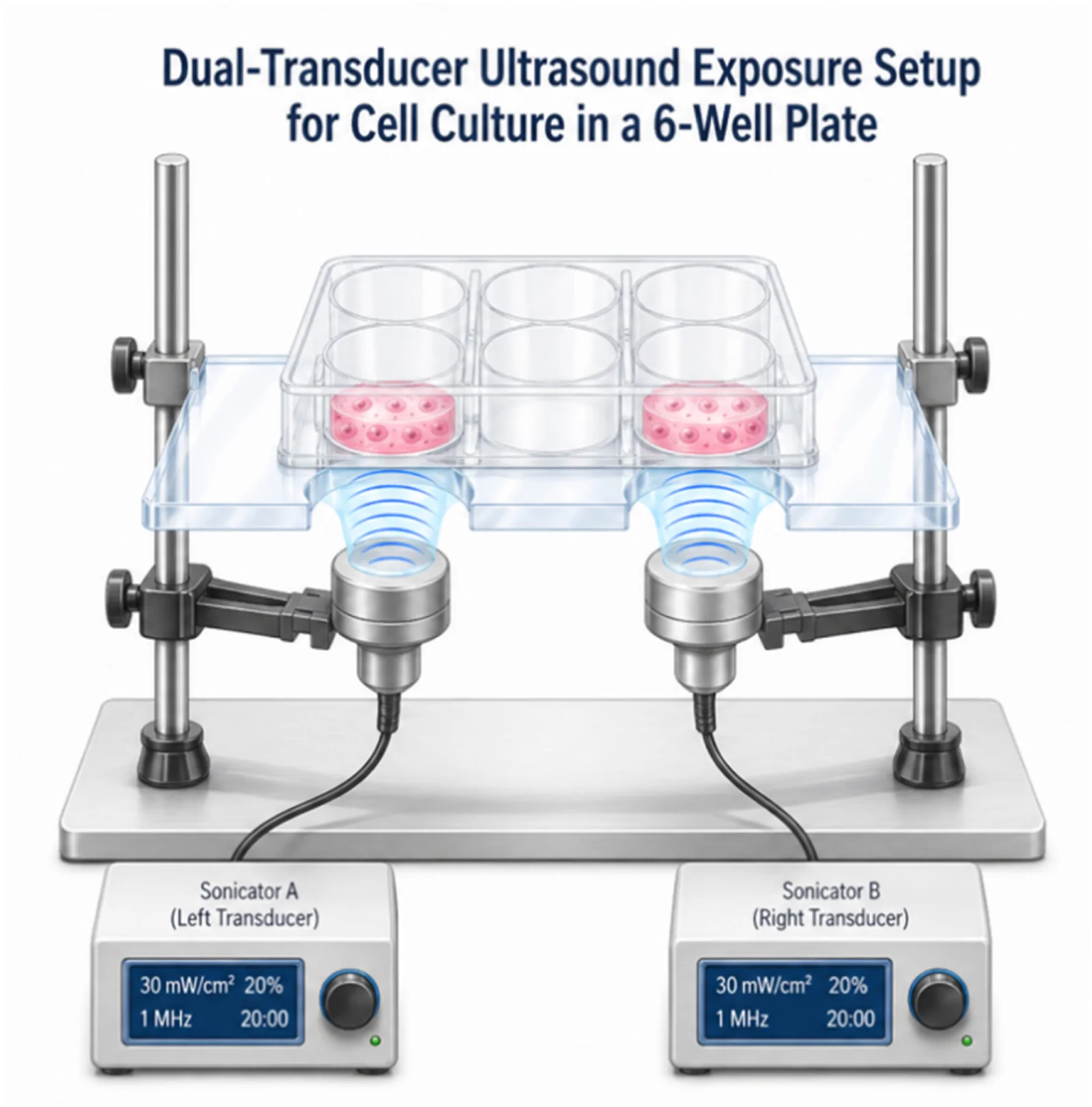
In vitro custom LIPUS treatment setup. Schematic of the configuration used to deliver low-intensity pulsed ultrasound to MC3T3-E1 cultures. Cells were grown as a monolayer on the base of the culture plate. The ultrasound transducer was positioned 6 mm directly beneath the plate and coupled through a layer of acoustic coupling gel to maintain continuous acoustic contact and eliminate air gaps that would reflect the wave.

Two 1 MHz ultrasonic transducers (ME electronics) were installed in an acrylic sheet with cutouts to ensure repeatable alignment. To minimize attenuation, culture plates were placed approximately 6 mm above the transducer faces using gel coupling. The LIPUS signal was administered using a therapeutic ultrasound unit (Sonicator 740, ME electronics) with the following parameters: 30 mW/cm^2^ spatial-average temporal-average intensity, 100 Hz pulse repetition frequency, 20% duty cycle, 1 MHz frequency, and 20 min per session. Sessions for differentiation experiments were held on the indicated treatment days between 0 and 14 days of culture (Fig. 17).

### 4.4 Cell proliferation assay (MTS)

Cell proliferation was assessed using the CellTiter 96 AQueous One Solution Cell Proliferation Assay (MTS; Promega). 10,000 cells were seeded per well in 96-well plates. At each time point, MTS reagent was applied to each well in accordance with the manufacturer's procedure, and absorbance was measured at 490 nm with a microplate reader (BioTek). Background absorbance from medium-only wells was subtracted, and the results were standardized to the Day 1 control. Data are presented as a percentage of Day 1 control. Four wells were studied for each condition and time point.

### 4.5 Alkaline phosphatase (ALP) staining

Cells were rinsed three times with PBS and briefly fixed with 4% paraformaldehyde before being stained with BCIP/NBT Liquid Substrate System (Sigma-Aldrich) on days 7 and 14. After approximately 60 min, the wells were rinsed with PBS until clear. Images were obtained with an upright light microscope. ImageJ's usual quantification workflow included converting to 8-bit, subtracting background (50μm, rolling ball), setting a global threshold, binarizing to a mask, filling holes, and measuring stained area fraction. The stain area within each field of view was computed. 5 fields per sample were imaged and analyzed.

### 4.6 Wound-healing migration assay

Migration was examined using 35 mm ibidi Culture-Insert 2 Well dishes (ibidi, Germany) in accordance with the manufacturer's protocol. 70 μL of cell suspension at 7 × 10^5^ cells/mL (control or TMEM63A-knockdown) was introduced to each insert well and left to adhere overnight. The following day, inserts were carefully removed using sterile forceps to leave a defined cell-free space of approximately 800 μm. Dishes were gently rinsed with PBS to remove non-adherent cells. Fresh osteogenic induction media was then added. LIPUS-treated dishes received a single 20-min LIPUS session immediately following gap formation. A light microscope was used to obtain phase-contrast photographs of the gap region at 0, 4, 8, 12, and 24 h after it had been created. Cell-covered area within the standardized region of interest was quantified using ImageJ, and percent cell-covered area was calculated as the fraction of the original gap region occupied by migrating cells at each time point. Wound closure was quantified as scratch width as well, to remove the dependence on baseline cell coverage. At each of 5 evenly spaced horizontal positions per field, width was taken as the longest contiguous span between the fronts of the continuous cell sheet, and the 5 values were averaged to give a single width per field. Two fields were averaged per sample, giving n = 3 samples per group at each timepoint. Width at each time point was normalized to the width of the same sample at 0 h. Migration velocity was calculated as (W_0_ − W_t_)/2t, where W_0_ is the width at 0 h, W_t_ is the width at time t, and the factor of two accounts for closure from both wound fronts.

### 4.7 RNA isolation and qRT-PCR

Total RNA was obtained using the TRIzol technique (Invitrogen) in accordance with the manufacturer's instructions. Cells were lysed in 1 mL TRIzol reagent per well of a 6-well plate, incubated at room temperature for 5 min, then combined with 200 μL chloroform, vortexed for 15 s, then centrifuged at 12,000 × g for 15 min at 4°C. After transferring the aqueous phase to a clean tube, RNA was precipitated with 500 μL of isopropanol, washed twice with 75% ethanol, air-dried, and resuspended in nuclease-free water. RNA content and purity were determined using a NanoDrop spectrophotometer. RNA was reverse-transcribed to cDNA, and qPCR used SYBR chemistry with gene-specific primers. Reference gene: β-actin. Relative expression was computed via 2^−ΔΔCt. Target panels included Tmem63a, Runx2, Alp, Osx, Col1a1, and β-actin (Table 1). Melt curves verified amplicon specificity. All qRT-PCR experiments were performed in technical triplicate from three independent biological replicates.

**Table 1:** Primer sequences used for RT-PCR experiments.

Gene Name	Forward primer sequence	Reverse primer sequence
Tmem63A	CTGTCTTCAGCGTCATCTAC	GTTTGTCTCACCAGACTCTC
Runx2	GCCACTTACCACAGAGCTATT	GAACAAACTAGGTTTAGAGTCATCAAG
Col1a1	CCAATGGTGCTCCTGGTATT	GGTTCACCACTGTTACCCTT
Alp	CCAACTCTTTTGTGCCAGAGA	GGCTACATTGGTGTTGAGCTTTT
Osx	ATGGCGTCCTCTCTGCTTG	TGAAAGGTCAGCGTATGGCTT
β-actin	GAGCTATGAGCTGCCTGACG	AGTTTCATGGATGCCACAGG

### 4.8 Immunoblotting

Immunoblotting was performed using the fully automated Abby capillary Western system (ProteinSimple, San Jose, CA) according to the manufacturer's instructions. Briefly, total protein lysates from cells were quantified using the BCA assay, and equal amounts of protein were loaded directly into the Abby instrument using the supplied chemiluminescent detection cartridge. The instrument automatically carried out protein separation, immunoprobing, washing, and signal detection. The following primary antibodies were used for immunoblotting analysis: Rabbit anti-Tmem63A, rabbit anti-pAKT, rabbit anti-AKT and anti-β-actin (loading control) (ProteinTech, Rosemont, IL). HRP-conjugated goat anti-rabbit secondary antibodies (Sigma) were used at 1:5000 dilutions. Protein bands were visualized and quantified automatically using Compass for Simple Western software (ProteinSimple). Peak area integration was used for quantitative analysis, and target protein levels were normalized to β-actin or total protein signal to correct for loading variability.

### 4.9 B-catenin nuclear translocation assay

The cells were plated at 120,000 cells per dish in MatTek glass-bottom microwell dishes (35-mm Petri dish, MatTek, Ashland, MA) and maintained at 37°C and 5% CO_2_. After 90% confluency, the cells were treated with or without LIPUS stimulation for 20 min, and fixed with 4% paraformaldehyde 5 min post treatment. After permeabilization with 0.1% Triton X-100, blocking with 5% BSA, cells were stained with anti-β-catenin primary antibody (1:1000), followed by goat anti-rabbit IgG H&L (Alexa Fluor 488) fluorescent secondary antibody. Nuclei were counterstained with DAPI. Images were acquired using identical exposure and gain settings over atleast 3 ROIs in each sample using a Zeiss Axiovert 200 M (LSM 510 META) laser scanning confocal microscope (Carl Zeiss, Germany). Quantification used ImageJ/Fiji: nuclear ROIs were segmented from DAPI; cytoplasmic ROIs were segmented after subtracting the nuclear ROIs. β-catenin intensity was measured in each ROI and expressed as a nuclear/cytoplasmic (N/C) intensity ratio. Images were analyzed across 3 independent experiments, and comparisons were evaluated with *t*-test or ANOVA.

### 4.10 Calcium imaging

Control and TMEM63A-knockdown MC3T3-E1 cells were seeded in 50-mm glass-bottom dishes (Mattek) at 100,000 cells per dish and allowed to attach for 24 h. The cells were stained using 2 mL of 5 μM CalciFluor™ Fluo-4 AM for 30 min at 37°C and 5% CO_2_ and then 30 min at room temperature. The cell dishes were shielded from light in aluminum foil. After incubation, the cells loaded with CalciFluor™ Fluo-4 AM were visualized and imaged to observe calcium oscillations using a Zeiss Axiovert 200 M (LSM 510 META) laser scanning confocal microscope, with FITC excitation/emission filters. Live Ca^2+^ imaging was performed on the inverted confocal microscope with the objective beneath the culture dish. The ultrasound transducer was mounted above the dish on a lab clamp and lowered into the culture medium so that its emitting face was submerged, positioned 6 mm above the cell monolayer and coaxial with the imaging field. The culture medium served as the coupling medium; no additional gel was used. The total time of the calcium oscillation experiment was 6 min, including 1 min at baseline without LIPUS stimulation, 3 min of active stimulation, and 2 min regression. Time-lapse sequences were collected every 4 s for 6 min. We found that not all the cells were sensitive to LIPUS stimulation. Therefore, we chose five active Ca^2+^ oscillating cells within the field of view for the image analysis. In all groups in this experiment, the fluorescence intensities of five cells in each field were quantified using ImageJ. The Ca^2+^ signal was calculated as ΔF/F_0_, where F_0_ is the mean baseline fluorescence intensity for the 1 min pre-stimulation period and ΔF is the deviation at each subsequent timepoint.

### 4.11 Bulk RNA sequencing

For RNA-seq, control and TMEM63A-knockdown MC3T3-E1 cells were cultured in osteogenic induction medium with or without daily LIPUS stimulation as described above. At Day 7 and Day 14 of differentiation, total RNA was extracted using the TRIzol method as described above (n = 3 biological replicates per condition; 12 samples total). RNA integrity was assessed using a Bioanalyzer 2100 (Agilent Technologies); only samples with RIN ≥ 7.0 were used for sequencing. Library preparation, sequencing, and bioinformatic analysis were performed by Novogene Corporation (Sacramento, CA). For RNA sequencing library preparation, messenger RNA (mRNA) was enriched from total RNA using poly-T oligo-attached magnetic beads. Purified mRNA was fragmented and reverse transcribed into first-strand cDNA using random hexamer primers, followed by second-strand cDNA synthesis. Libraries were subsequently subjected to end repair, A-tailing, adapter ligation, size selection, PCR amplification, and purification, and were quantified using Qubit fluorometry and real-time PCR, with fragment size distribution assessed on the Bioanalyzer. Following quality control, libraries were pooled and sequenced on an Illumina platform using sequencing-by-synthesis chemistry to generate transcriptome sequencing data. Raw RNA-sequencing reads were subjected to quality assessment using FastQC (v0.12.1). High-quality reads were aligned to the mouse reference genome (GRCm39) using STAR (v2.7.10b), and gene- and transcript-level abundances were quantified with RSEM (v1.2.28).^37^ Transcript abundance estimates were imported into R using the tximport package and analyzed for differential gene expression using DESeq2. Differential expression analysis was performed using a negative binomial generalized linear model framework, with Wald tests used to assess statistical significance. Resulting p-values were adjusted for multiple hypothesis testing using the Benjamini-Hochberg false discovery rate (FDR) correction method. Gene Set Enrichment Analysis (GSEA, v4.3.2) was performed to identify significantly enriched biological pathways using gene sets from the Molecular Signatures Database (MSigDB v7.4).^38^ Genes were ranked based on differential expression statistics incorporating both fold change and significance, and enrichment was assessed against the Hallmark and Reactome gene set collections. Enrichment scores were calculated using a weighted statistic with 1000 permutations to estimate statistical significance. Gene sets containing fewer than 15 genes or more than 500 genes were excluded from the analysis.

### 4.12 Software and reproducibility

Data analysis used GraphPad Prism and Fiji/ImageJ.

### 4.13 Experimental design and statistics

Biological replicates were n = 3 per condition unless stated. Data are reported as mean ± SD. For two groups, unpaired two-tailed t-tests were used; for multi-factor comparisons, two-way ANOVA with Tukey post-hoc tests. Significance thresholds: p < 0.05 (∗), <0.01 (∗∗), <0.001 (∗∗∗), <0.0001 (∗∗∗∗).

## CRediT authorship contribution statement

**Meghana Davuluri:** Writing – review & editing, Writing – original draft, Visualization, Validation, Methodology, Formal analysis, Data curation, Conceptualization. **Rekha Sathian:** Software, Formal analysis, Data curation. **Anna R. Cho:** Validation, Methodology. **Ramana V. Davuluri:** Validation, Software, Resources, Formal analysis. **Yi-Xian Qin:** Writing – review & editing, Writing – original draft, Visualization, Validation, Supervision, Software, Resources, Project administration, Methodology, Investigation, Funding acquisition, Formal analysis, Conceptualization.

## Data availability

All data supporting the findings are available from the corresponding author upon reasonable request. Image analysis macros and raw data can be provided.

## Ethical Approval

This study does not contain any studies with human or animal subjects performed by any of the authors.

## Declaration of competing interest

The authors declare that they have no known competing financial interests or personal relationships that could have appeared to influence the work reported in this paper.
